# Collaborative Design and Development of a Patient-Centered Digital Health App for Supportive Cancer Care: Participatory Study

**DOI:** 10.2196/73829

**Published:** 2025-11-11

**Authors:** Sonia Difrancesco, Matthia Martina Bauert, Claude Lehmann, Steven Häsler, Yi Zhang, Sven Hirsch, Philipp Ackermann, Kurt Stockinger, Monika Reif, Sunjoy Mathieu, Anna Götz, Andreas Wicki, Michael Krauthammer, Claudia M Witt

**Affiliations:** 1 Department of Quantitative Biomedicine University of Zurich Zurich Switzerland; 2 Department of Health Science and Technology ETH Zurich Zurich Switzerland; 3 Institute of Computer Science ZHAW Zurich University of Applied Sciences Winterthur Switzerland; 4 Institute of Computational Life Sciences ZHAW Zurich University of Applied Sciences Wädenswil Switzerland; 5 Institute of Applied Mathematics and Physics ZHAW Zurich University of Applied Sciences Winterthur Switzerland; 6 Department of Medical Oncology and Hematology University Hospital Zurich and University of Zurich Zurich Switzerland; 7 Institute for Complementary and Integrative Medicine University Hospital Zurich and University of Zurich Zurich Switzerland

**Keywords:** cancer, digital health, mHealth, patient-reported outcome measures, supportive care

## Abstract

**Background:**

Digital health tools such as smartphone apps have the potential to improve supportive cancer care. Although numerous smartphone apps for supportive care are available, few are designed using a user-centered approach. Such an approach is crucial for successful implementation, as it may improve user engagement, usability, and adoption in clinical settings.

**Objective:**

This study aimed to co-design and develop a digital health app for supportive cancer care in collaboration with patients with cancer and health care professionals and to explore factors influencing its future acceptance.

**Methods:**

We conducted a participatory study with the major stakeholders at the University Hospital Zurich. Workshops, individual qualitative interviews, and focus groups were held with health care professionals, survivors of cancer, and patients with cancer. The co-design process was divided into 3 phases: predesign, generative phase, and prototyping. User-centered design methods included scoring cards and think-aloud protocols to co-create design ideas, identify important functionalities, and test usability. Qualitative data were analyzed using thematic analysis.

**Results:**

Patients and health care professionals emphasized the need for a digital health app to improve patient–healthcare professional communication, digitalize supportive care screening and processes, and enhance self-efficacy. The resulting app, OncoSupport+, was co-designed and integrated into the clinical workflow for supportive cancer care. It consists of (1) a patient dashboard to record patient-reported outcome measures and to provide access to personalized supportive care information and contact details, and (2) a nurse dashboard to visualize patient data, which can be used during nursing consultations. Potential facilitators for adoption included ease of use, workflow integration, introduction by health care professionals, and technical support, whereas internet anxiety may be a potential barrier.

**Conclusions:**

Collaborative development with patients and health care professionals is crucial for creating digital health tools that can be implemented successfully. Future research should evaluate the feasibility of long-term implementation and the real-world usability and effectiveness of OncoSupport+ for improving communication, self-efficacy, and quality of life.

## Introduction

Cancer is a highly prevalent condition with increasing incidence rates and is the leading cause of death worldwide [1]. As improvements in diagnosis, treatment, and surveillance have led to better survival rates, patients with cancer are living longer [2], though a considerable portion of patients experience significant morbidity and symptoms related to both the cancer and its treatment. Patients with cancer often exhibit physical, emotional, social, spiritual, and informational needs, also known as supportive care needs, and the aggregated impact may ultimately contribute to exacerbated health outcomes and diminished quality of life [3]. Supportive care, which focuses on the prevention and management of cancer symptoms and cancer treatment–related symptoms, has been widely acknowledged as an essential component of comprehensive cancer care [4]. It includes the management of physical and psychological symptoms and side effects across the cancer care trajectory from diagnosis through treatment to posttreatment care. Examples of supportive care include management of symptoms such as nausea, nutritional and exercise support, psychological support, and practical assistance for concerns such as transportation [4].

Several cancer centers have established specialized supportive care services, employing the expertise of a multidisciplinary team with diverse medical specializations such as nutritionists, physiotherapists, and psychologists. Supportive care has been shown to improve quality of life [5], tolerability of cancer treatments, survival, and systemic health economic benefits [6]. However, studies continue to report unmet supportive care needs, such as physical and informational needs, indicating that significant efforts are still required to ensure the effective implementation of evidence-based supportive care in clinical practice [7]. Specifically, the Multinational Association of Supportive Care in Cancer has emphasized the routine collection of patient-reported outcome measures (PROMs) to facilitate timely, individualized supportive care and empower patients through evidence-based education [7]. Commonly used PROMs include the European Organization for Research and Treatment of Cancer Quality of Life Questionnaire Core 30 (EORTC QLQ-C30) [8] and the National Comprehensive Cancer Network (NCCN) Distress Thermometer [9]. In this context, digital health tools, particularly mobile health (mHealth) apps, have emerged as promising solutions for delivering patient-centered supportive care interventions [10].

A growing number of digital health applications offer mental health support, symptom self-management, and educational resources. Among these, digital symptom monitoring and electronic patient-reported outcome measures (ePROMs) are commonly integrated interventions for supportive cancer care [11,12] and provide advantages over traditional paper-based PROMs or retrospective questionnaires, which are often limited by recall bias and low completion rates [13,14] . As digital health apps enable the remote collection of symptoms and supportive care needs in daily living environments, these tools allow for a more timely and accurate assessment of patient well-being. Research has shown that such applications can improve communication with health care professionals, facilitate connections with other patients, and enhance self-efficacy, thereby complementing clinical care [15]. Systematic reviews and meta-analyses have further suggested that digital health interventions may improve symptoms and quality of life [11,15]. Despite these findings, their implementation and adoption remain limited, particularly in cancer care [16]. A key reason is the limited involvement of key stakeholders, including patients and health care professionals, during development, leading to suboptimal adoption and effectiveness [10,11,17].

Co-design has been identified as a promising approach to ensure that digital health interventions are patient-centered, clinically relevant, and technically feasible [18]. By incorporating participatory and iterative stakeholder engagement, co-design can help tailor digital health interventions to meet the needs and preferences of both patients and health care providers while facilitating the early identification of implementation barriers and factors influencing adoption and long-term engagement. However, despite its potential, co-design is not always rigorously applied in digital health research, as studies often lack structured methodologies, fail to systematically integrate diverse stakeholder input, and do not adequately assess long-term feasibility [19]. Addressing these gaps is crucial for developing user-centered and sustainable digital health interventions in supportive cancer care.

The overall aim of this study was to collaboratively design and develop a digital health app for supportive cancer care together with the relevant stakeholders (patients with cancer, survivors of cancer, and health care professionals). Specifically, the first objective was to map the context of supportive cancer care at the University Hospital Zurich, including challenges and needs. The second objective was to identify specific functionalities and features of the app and gather the patients’ and nurses’ perspectives; as part of this objective, we also explored factors that can potentially influence the uptake and implementation of the digital health app for supportive cancer care. Finally, the third objective was to design and develop the prototype and user interface of the digital health app to be technically implemented.

## Methods

### Ethical Considerations

This study was reviewed by the Ethics Committee of the Canton of Zurich, which determined that it did not fall within the scope of the Swiss Human Research Act and therefore did not require formal approval (Req-2023-01095). According to the Swiss Human Research Act (SR 810.30), ethical approval is required for studies involving health-related personal data, biological material, or clinical interventions. This study involved collecting opinions and experiences from patients, advocates, and nurses to inform app development; no health-related personal data were analyzed. Patients, patient advocates, and nurses received written and oral information about the study objectives and procedures, and written informed consent was obtained before participation. Data were collected in a pseudonymized manner. Transcripts and digital records were deidentified before analysis, and all personal identifiers were removed. Access to study data was restricted to the research team. As patient advocates were part of Swiss Group for Clinical Cancer Research (SAKK), they received financial compensation for their contribution. No quotes or materials that could identify individual participants are included in the manuscript or supplementary materials.

### Study Design

This participatory study was conducted between May 2023 and October 2024 as part of the Digital Health Zurich initiative [20]—a collaboration between the University of Zurich, the University Hospital of Zurich, and the Zurich University of Applied Sciences. The overarching goal of Digital Health Zurich is to create a sustainable, data-driven, and cross-institutional digital ecosystem by fostering collaboration between patients, health care professionals, technology developers, and researchers in Zurich and beyond. The initiative aims to translate research into real-world digital health solutions, with a strong focus on patient and provider needs, participatory development, cross-institutional cooperation, and innovation within the health care system. As a use case, this study was conducted at the University Hospital Zurich in collaboration with the Department of Oncology and Hematology and the Comprehensive Cancer Center Zurich.

**Figure 1:**
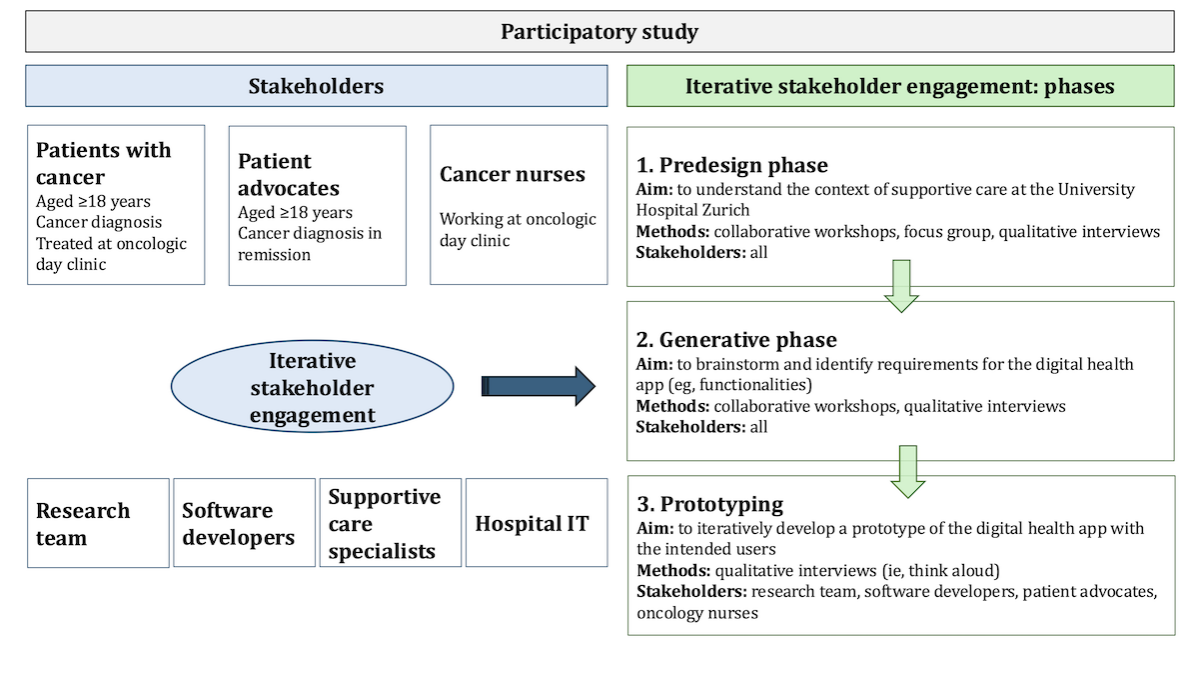
Participatory study design using iterative stakeholder engagement, divided into 3 phases: predesign, generative, and prototyping.

The stakeholders included in the study were patients with cancer and patient advocates, cancer nurses, supportive care specialists, oncologists, and the research team; more details are listed in the Study Participants and Stakeholders section. Figure 1 shows an overview of the study design, using iterative stakeholder engagement, user-centered approaches, and design thinking techniques. The study can be divided into different phases, similar to the framework suggested by Noorbergen et al [18] for co-designing mHealth systems, which is a novel extension of the work by Sanders and Stappers [21]. The predesign phase aimed to understand the context of supportive cancer care at the University Hospital of Zurich, including the challenges faced by cancer nurses and patients in accessing supportive cancer care. The generative phase focused on brainstorming digital health app functionalities, as well as identifying potential factors that may impact future uptake of the technology. The prototyping phase, taking into account the findings from the previous phases, aimed to iteratively develop a prototype of the digital health app by gathering feedback from the intended users. Between June 2023 and October 2024, the research team held collaborative workshops, focus groups with cancer nurses, and qualitative interviews with patients and patient advocates.

### Study Participants and Stakeholders

#### Patients With Cancer and Patient Advocates

Patients with cancer and patient advocates were recruited for the study. Inclusion criteria for patients with cancer were: (1) current treatment for cancer at the oncologic day clinic of the Department of Oncology and Hematology, (2) age ≥18 years, and (3) ability to speak German, Italian, or English. As patients with cancer often experience fatigue and may face challenges in participating in research activities, patient advocates from the SAKK (today Swiss Cancer Institute) were also included. These advocates were survivors of cancer in remission, enabling them to contribute insights from both patient and survivor perspectives. Their ability to engage more actively made them valuable contributors to the research. Inclusion criteria for patient advocates were (1) history of cancer, (2) age ≥18 years, and (3) ability to speak German, Italian, or English.

#### Cancer Nurses

Cancer nurses were included in the study. Inclusion criteria were employment at the Department of Oncology and Hematology of the University Hospital Zurich.

#### Supportive Care Specialists

Supportive care professionals, including a supportive care nurse, a nutritionist, 2 physiotherapists, and medical doctors with specializations in oncology, palliative care, and complementary medicine, were engaged in the participatory study to share their expertise and ensure that the app aligned with clinical practices in supportive cancer care.

#### Research Team

The research team consisted of one cancer nurse and one oncology doctor, researchers in digital health, computer science, computational health, and medical informatics, as well as a user experience designer. Two of the computer science researchers also contributed as software developers for the digital health app. Digital health researchers were responsible for organizing, moderating, and conducting the research activities. One of the computer science researchers supported data collection.

### Recruitment of Patients With Cancer, Patient Advocates, and Cancer Nurses

Patients with cancer were recruited by cancer nurses from the Department of Oncology and Hematology at the University Hospital Zurich. Potential participants were introduced to the study through an informational flyer describing the study procedures and offering the option to participate in interviews while receiving systemic antineoplastic therapy at the oncologic day clinic. Interested patients could provide their contact details and suggest a convenient date and time for an interview with a researcher. Of the 8 patients who provided their contact details, a total of 6 agreed to participate in the study. Patient advocates were identified by the coordinator of patient advocates at SAKK, and 4 agreed to participate. A total of 5 nurses were recruited by a project team member who also works as an oncology nurse at the day clinic.

### Study Procedure

Between June 2023 and October 2024, data were collected through collaborative workshops, focus groups with oncology nurses, and qualitative interviews with patients with cancer and patient advocates. Interviews and focus groups were conducted in German, English, or Italian, while workshops were held in English, the working language of the project team. Focus groups and interviews were audio-recorded, whereas workshops were documented by 2 researchers who took notes, cross-checked them, and shared the notes with the wider team.

#### Phase 1: Predesign

The predesign phase consisted of a collaborative workshop with the research team, one focus group with cancer nurses, and individual qualitative interviews with patients and patient advocates. The initial workshop aimed to map the context of supportive cancer care at the hospital and define the study problem statement. The subsequent 1.5-hour focus group with cancer nurses was guided by questions on their experiences with supportive care, challenges in daily practice, and opportunities for digital health to enhance supportive care. In parallel, interviews with patients with cancer and patient advocates explored their experiences with supportive care services and their views on how digital health tools could support their needs.

#### Phase 2: Generative

The generative phase included a second collaborative workshop with the research team, qualitative interviews with patients with cancer and patient advocates, and a workshop with supportive care specialists. Informed by the predesign phase, the research team specified potential functionalities for integration into the app. During the interviews, patients and patient advocates were asked to reflect on potential app functionalities, suggest additional features, and discuss factors that might influence the uptake of digital health interventions. To prioritize functionalities, a scoring exercise was included in which participants rated each feature on a scale from 1 to 10, with 10 indicating a “must-have” functionality. The collaborative workshop with the research team focused on brainstorming digital health solutions and proposing an initial structure for the app. As the preceding step highlighted the need to personalize supportive care for patients with cancer, a 2-hour workshop was conducted with supportive care specialists and digital health researchers, during which participants co-designed a rule-based algorithm to tailor supportive care.

#### Phase 3: Prototyping

From July to October 2024, the prototyping phase was conducted. Based on insights from the previous phases, a digital health researcher iteratively developed user requirements and specifications documented in Confluence [22], which were then translated into user interface designs with Figma [23] by the user experience designer. A user flow was created to outline app navigation, and this flow was tested by 2 cancer nurses and 4 patient advocates. The prototype of the user interface was subsequently developed and iteratively reviewed by the same group. Participants were encouraged to verbalize their impressions, identify challenges in navigation, and comment on the clarity and usefulness of the proposed functionalities. Finally, a workshop involving health care professionals and the research team was held to evaluate and further refine the prototype.

A meeting between the software developers and the IT department of the hospital was also held to assess different digital solutions and explore how these could potentially be integrated into the IT system of the hospital.

### Data Analysis

The qualitative data from interviews and focus groups were transcribed, translated, and analyzed using thematic analysis, following Braun and Clarke [24] methodology.

All audio recordings were transcribed orthographically using OpenAI Whisper [25], performed locally to ensure data security. Transcripts were translated to English, initially using Whisper and then manually checked and corrected by bilingual researchers who conducted the interviews, ensuring high-quality transcription and translation. The corrected transcripts were used for analysis. Thematic analysis was conducted following the six-step process [24]: (1) familiarization with the data, (2) generating codes, (3) searching for themes, (4) reviewing themes, (5) defining and naming themes, and (6) producing a report. To reduce bias, analyses were conducted by the first 2 coauthors of this study. During familiarization, the first 2 coauthors read transcripts multiple times and highlighted text segments relevant to 5 aspects: app and dashboard functionalities, barriers and facilitators to use, challenges with the health care system and supportive care, and experiences with digital devices. Initial codes were generated using an open coding approach, capturing meaningful segments without predefined categories. Codes were then grouped based on similarity to identify patterns and inform preliminary theme development.

Themes were iteratively reviewed, refined, and organized into coherent subthemes, with disagreements resolved through discussion between the first 2 coauthors. Theme names were defined to accurately reflect core concepts, and data extracts were used to support each theme. Results were summarized with mind maps, journey maps, and frequencies of reported themes. Median and ranges were reported to summarize scorecards on functionalities.

### Unified Theory of Acceptance and Uptake of Technologies

As it is important to identify factors influencing acceptance early in the design phase to guide design choices and improve engagement, the Unified Theory of Acceptance and Use of Technology (UTAUT) framework was used to guide reporting of the results [26]. Originally developed to predict technology acceptance in organizational settings, the framework has been validated in health care contexts [27] and adapted for mHealth adoption in cancer care [28]. The validated framework by Philippi et al [28] identifies 3 key predictors of acceptance: performance expectancy, effort expectancy, and social influence. Internet anxiety moderates their effects.

Performance expectancy refers to the extent to which an individual believes the technology will be beneficial and is considered the strongest predictor of acceptance. Effort expectancy describes the perceived ease of use of the system, with higher effort expectancy (ie, lower ease of use) negatively impacting acceptance. Social influence refers to the degree to which users perceive that significant others encourage system usage, positively influencing adoption. Internet anxiety moderates the relationship between effort expectancy and social influence, potentially hindering adoption. Specifically, the UTAUT was used to report the identified themes related to barriers and facilitators to uptake.

### Sample Size and the Principle of Saturation

Although the study initially aimed to enroll approximately 30 participants for qualitative interviews and focus groups, the principle of thematic and design saturation was applied [29]. Recruitment was stopped once no new themes emerged, ensuring that data collection was both rigorous and meaningful without overburdening participants. For instance, when 2 or more consecutive interviews or focus groups did not reveal new themes or suggest new design features, the recruitment was stopped. This approach is consistent with co-design studies in digital health, where sample sizes are often small and vary widely; a recent umbrella review reported that as few as 2 participants have been included in some co-design projects [30]. In addition, as mentioned by Arcia at al [31] what matters most is not the absolute number of participants, but the iterative design process and achievement of thematic and design saturation, both of which were ensured in this study.

## Results

### Demographics, Experience With Digital Tools, and Other Characteristics of Patients With Cancer, Patient Advocates, and Cancer Nurses

A total of 6 patients with cancer and 4 patient advocates participated in the study (Table 1). Their ages ranged from 29 to 75 years, with a mean of 50.3 years. Four participants were female. Most held advanced university degrees, with 6 out of 10 having completed a bachelor’s degree or higher (ie, master’s or PhD). None were employed full-time at the time of the study; 4 worked part-time, and 2 were retired. All participants owned both a smartphone and a computer. While all reported daily smartphone use, 7 also used a computer daily, and 6 owned a tablet. Half of the participants rarely needed assistance when using digital devices, 4 required help occasionally, and 1 needed assistance daily. All nurses who participated in the study were female, aged between 37 and 53 years, with at least 10 years of experience in oncology nursing.

**Table 1:** Sociodemographic characteristics, device ownership, daily use, and need for assistance with digital devices among patients with cancer and patient advocates (N=10).

Characteristic		Total sample (N=10)	Patient advocates (n=4)	Patients with cancer (n=6)
Age (years), mean (range)		50.3 (29-75)	51 (47-75)	47.5 (29-67)
**Sex, n (%)**				
	Female	4 (40)	1 (25)	3 (50)
	Male	6 (60)	3 (75)	3 (50)
**Education level, n (%)**				
	Primary education	0 (0)	0 (0)	0 (0)
	Secondary education	4 (40)	1 (25)	3 (50)
	Tertiary education	6 (60)	3 (75)	3 (50)
**Working status, n (%)**				
	Full time	0 (0)	0 (0)	0 (0)
	Part time	4 (40)	2 (50)	2 (33.3)
	Unable to work for health reasons	4 (40)	1 (25)	3 (50)
	Retired	2 (20)	1 (25)	1 (16.7)
	Unemployed	0 (0)	0 (0)	0 (0)
**Ownership of digital devices, n (%)**				
	Computer or laptop	10 (100)	4 (100)	6 (100)
	Smartphone	10 (100)	4 (100)	6 (100)
	Tablet	5 (50)	3 (75)	2 (33.3)
**Daily use of digital devices, n (%)**				
	Computer or laptop	7 (70)	4 (100)	3 (50)
	Smartphone	10 (100)	4 (100)	6 (100)
	Tablet	3 (30)	2 (50)	1 (16.7)
**Need for assistance when using digital devices, n (%)**				
	Rarely	5 (50)	2 (50)	3 (50)
	Sometimes	4 (40)	2 (50)	2 (33.3)
	Daily	1 (10)	0 (0)	1 (16.7)

### Phase 1 (Predesign): Supportive Care Pathway, Challenges, and Opportunities for Digital Health

**Figure 2:**
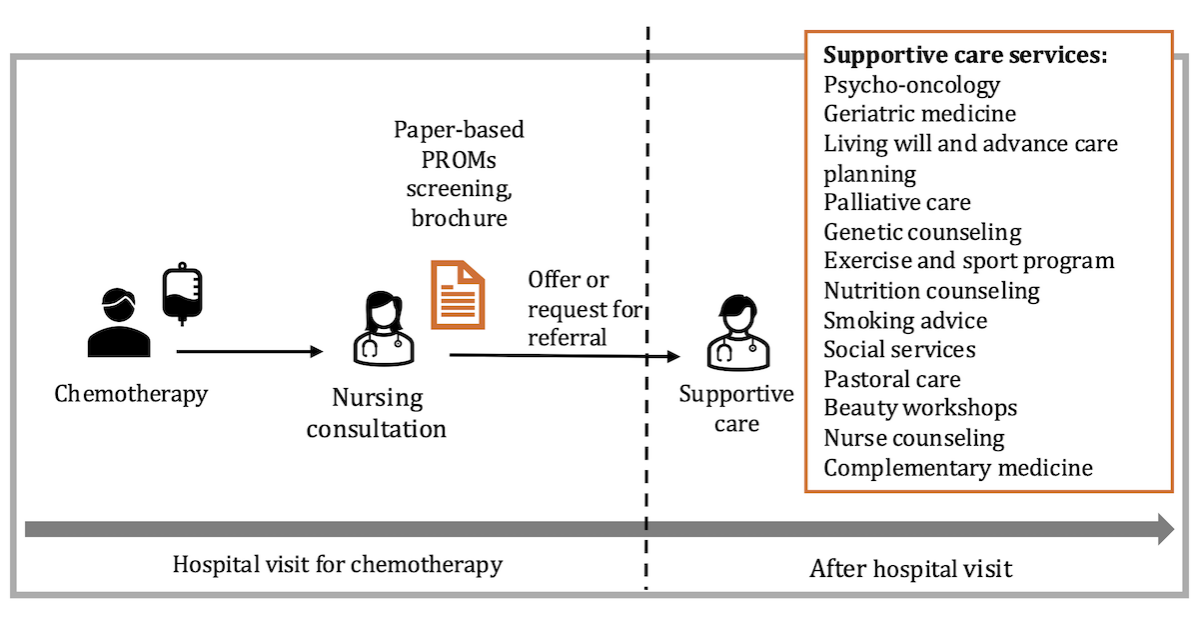
Mapping of supportive care pathways at the University Hospital Zurich.

Figure 2 shows the supportive care pathways at the University Hospital Zurich. Patients may access supportive care services through oncology nursing consultations, physician consultations, or direct contact. At the initial nursing consultation, supportive care needs are assessed using standardized paper-based PROMs, specifically the EORTC QLQ-C30 [8] and the NCCN Distress Thermometer [9]. Based on these assessments, nurses provide referrals to one or more of the 12 supportive care services offered, and patients additionally receive a supportive care brochure.

**Figure 3:**
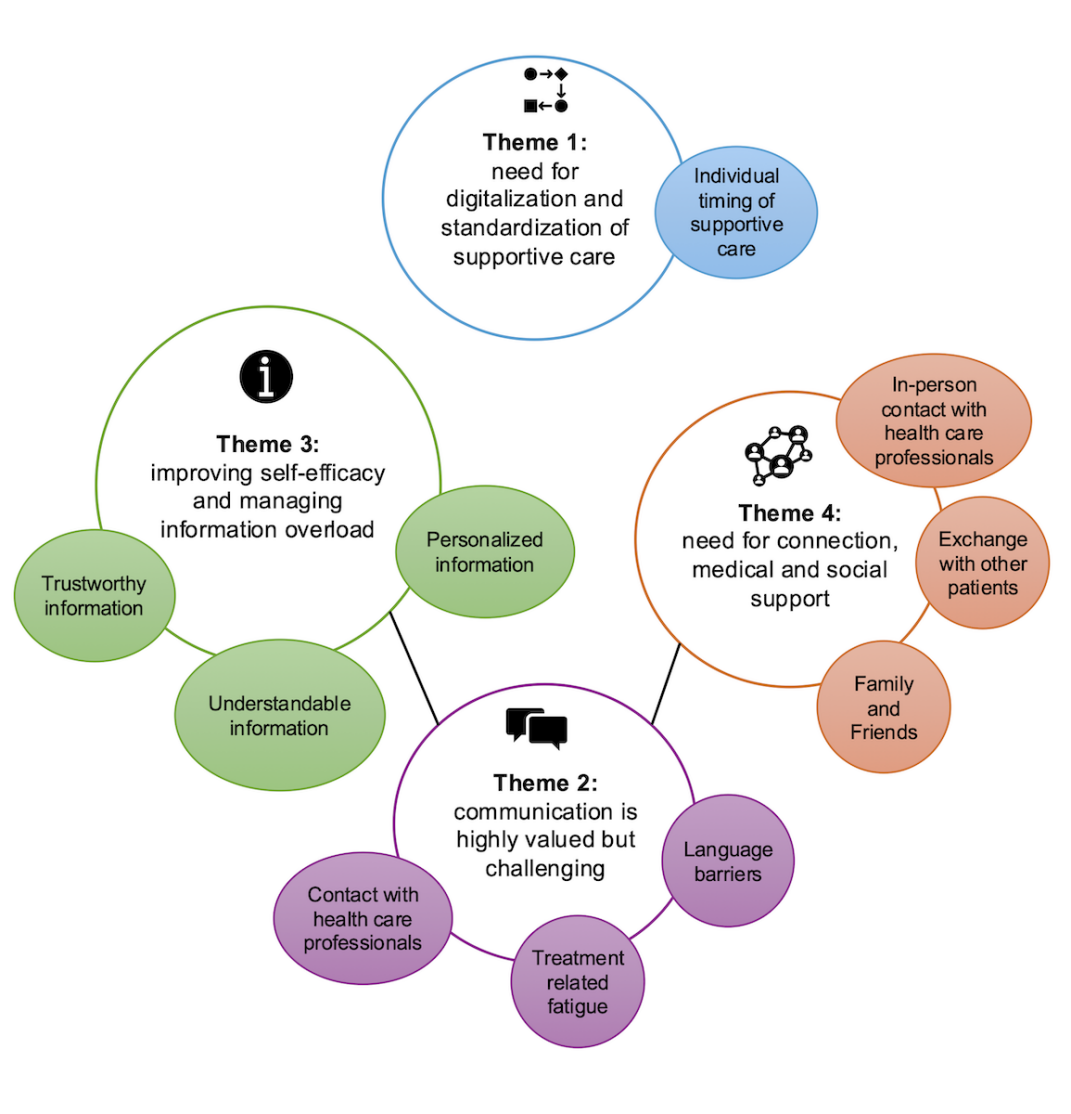
Challenges and opportunities for digitalization in supportive cancer care at the University Hospital Zurich. Themes were derived from interviews and focus groups with patients with cancer, patient advocates, and oncology nurses, and informed subsequent app functionalities.

The predesign phase revealed 4 major themes regarding challenges and opportunities for digitalization in supportive cancer care at the University Hospital Zurich (Figure 3). Nurses, patients, and patient advocates highlighted the need for digitalization and standardization of supportive care, suggesting that digital platforms could replace paper-based PROMs, improve screening rates, and allow consultations to focus on discussing patient responses. As one cancer nurse explained:

At the moment, we give patients a paper-and-pencil version of the screening when they come to the hospital. […] So, the digital screening should actually improve this by allowing patients to answer in advance […]. This way, patients can complete it at home, and we can spend more time discussing their responses during the consultation.

Cancer Nurse 2, focus group

Communication challenges were also emphasized, including difficulties in reaching doctors, treatment-related fatigue, and language barriers. One patient advocate noted:

It's difficult [the communication with healthcare professionals] ... most of the time, my oncologist answers quite quickly. But sometimes he has too many things to do.

Patient Advocate 3

Patients reported information overload, describing the volume of paper-based brochures and consultations as overwhelming, and expressed a preference for simplified, trustworthy, and personalized digital resources.

I have read a few things but, in the end, I was so overwhelmed with the conversations. Before each surgery, you have a conversation with the surgeon or the radiologist and then with the anesthetist and then again, a conversation and then you get a few more documents […]

Cancer Patient 6

Finally, the importance of connection and social support was highlighted. Patients valued peer support and digital opportunities to connect, but also emphasized that face-to-face consultations remain indispensable.

If I had an app for my illness, it would be good, but it would make everything so virtual, and you still need to connect with people in real life.

Cancer Patient 4

These themes and their relationships are summarized visually in Figure 3. More illustrative quotes can be found in Multimedia Appendix 1.

### Phase 2 (Generative): Functionalities and Integration in the Supportive Care Pathway

Table 2 summarizes the functionalities of the digital health app, named OncoSupport+, that were either proposed by the research team or suggested by patients, mapped to the related themes identified in phase 1 (Figure 3). Functionalities developed by the research team were rated by patients on a scale from 1 to 10, where higher scores indicated greater perceived importance. Personalized information on supportive care (median 9.5) and digital PROMs screening (median 9.0) were rated as the most relevant features. Other functionalities, including self-help group information, a diary for consultations, and PROMs visualization, also received favorable ratings (all medians ≥7). Patients suggested several additional functionalities, including patient–health care professional chat (proposed by 6 participants), patient–patient chat (5 participants), self-management advice and symptom checking (5 participants), proactive contact from health care professionals (4 participants), reminders (4 participants), sharing data with family and friends (3 participants), information about cancer types (3 participants), patient stories (3 participants), and supportive care information for relatives (2 participants). Several of these patient-suggested features were integrated into the app (eg, reminders, patient stories, cancer type information, and peer support links), while others were not implemented due to scope limitations or regulatory considerations.

**Table 2:** Proposed functionalities for OncoSupport+, by source (research team vs patients), with brief descriptions, related themes identified in the predesign phase (Figure 3), ratings by patients and patient advocates.

Functionality	Description	Related theme (phase 1)	Median (range)	Frequency of suggestions,n	Included (yes/no)
Digital PROMs^a^ screening^b^	Scheduled digital assessment of supportive care needs using standardized instruments: EORTC QLQ-C30^c^ and NCCN^d^ Distress thermometer (ePROMs^e^).	Digitalization and standardization; communication	9.0 (6-10)	—^f^	Yes
Personalized information on supportive care^b^	Personalized list of relevant supportive-care services and contact details generated from the patient profile and ePROMs results.	Information overload and self-efficacy	9.5 (7-10)	—	Yes
Self-help group information^b^	Link to verified cancer self-help and peer-support groups.	Information; connection, and peer support	7.5 (2-10)	—	Yes
Diary for consultations^b^	Free-text notes to record symptoms not captured by ePROMs and questions for nursing consultations.	Communication	7.5 (3-10)	—	Yes
PROMs visualization^b^	Dashboards for patients and cancer nurses displaying ePROMs and trends over time, with integration of diary notes.	Communication; self-efficacy	7.0 (2-10)	—	Yes
Patient–Health Care Provider chat^g^	Messaging between patients and cancer nurses.	Communication	—	6	No
Patient–patient chat^g^	Link to a peer-support platform provided by the cancer association.	Connection and peer support	—	5	Yes
Symptom checker and self-management advice^g^	Personalized symptom management advice based on reported symptoms and ePROMs results.	Self-efficacy	—	5	No
Proactive contact from health care provided (phone or email)^g^	Nurse-initiated outreach (phone or email) triggered by digital PROMs screening.	Communication	—	4	No
Appointment and PROMs reminders^g^	Automated reminders for clinic appointments and ePROMs completion by email, SMS, or mailed letter.	Self-efficacy	—	4	Yes
Sharing data with family and friends^g^	Patient-controlled sharing of selected PROMs summaries with family members and friends.	Social support	—	3	No
Information about cancer types^g^	Evidence-based educational content on cancer diagnoses, treatments, and common side effects.	Information overload; self-efficacy	—	3	Yes
Patient stories and experiences^g^	Positive stories and experiences from patients and survivors to support coping with cancer.	Emotional support and connection	—	3	Yes
Supportive care information for relatives^g^	Resources for relatives on available services and practical guidance.	Social support	—	2	No

^a^PROMs: patient-reported outcome measures.

^b^Research team suggestions.

^c^EORTC QLQ-C30: European Organization for Research and Treatment of Cancer Quality of Life Questionnaire Core 30.

^d^NCCN: National Comprehensive Cancer Network.

^e^ePROMs: electronic patient-reported outcome measures.

^f^Not available.

^g^Patient suggestions.

**Figure 4:**
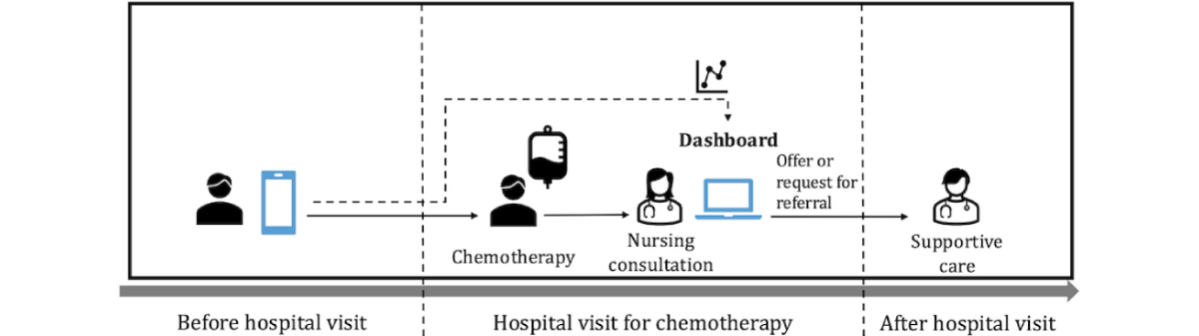
Integration of OncoSupport+ into the supportive care pathway.

Figure 4 summarizes how OncoSupport+ and the included functionalities are integrated into the supportive care pathway. Patients complete digital PROMs screenings (Multimedia Appendix 2 lists the entire digital screening questionnaires, including the EORTC QLQ-C30 and the NCCN Distress Thermometer tool) in the week before their consultation, after which the app provides personalized supportive care information and other more general information, such as self-help groups and the peer-support platform. During the consultation, nurses access a dashboard displaying PROMs, diary notes, and personalized information for supportive care and can recommend supportive care services. During the cancer treatment journey, patients use the app at least 3 times: at the beginning, halfway through treatment, and at the end.

To provide personalized supportive care information based on digital PROMs screening and the patient profile, a rule-based algorithm was developed in collaboration with supportive care specialists at the hospital. This algorithm prioritizes supportive care services and provides personalized information based on the symptoms and needs reported by patients and is based on medical knowledge and published scientific literature. For example, psycho-oncology is prioritized if a patient reports a distress score ≥5 on the NCCN Distress Thermometer and rates at least one emotional or social functioning item as “very much.” A detailed description of the algorithm and its rules is provided in Multimedia Appendix 3.

### Factors Potentially Impacting the Uptake of the Digital Health App OncoSupport+

**Figure 5:**
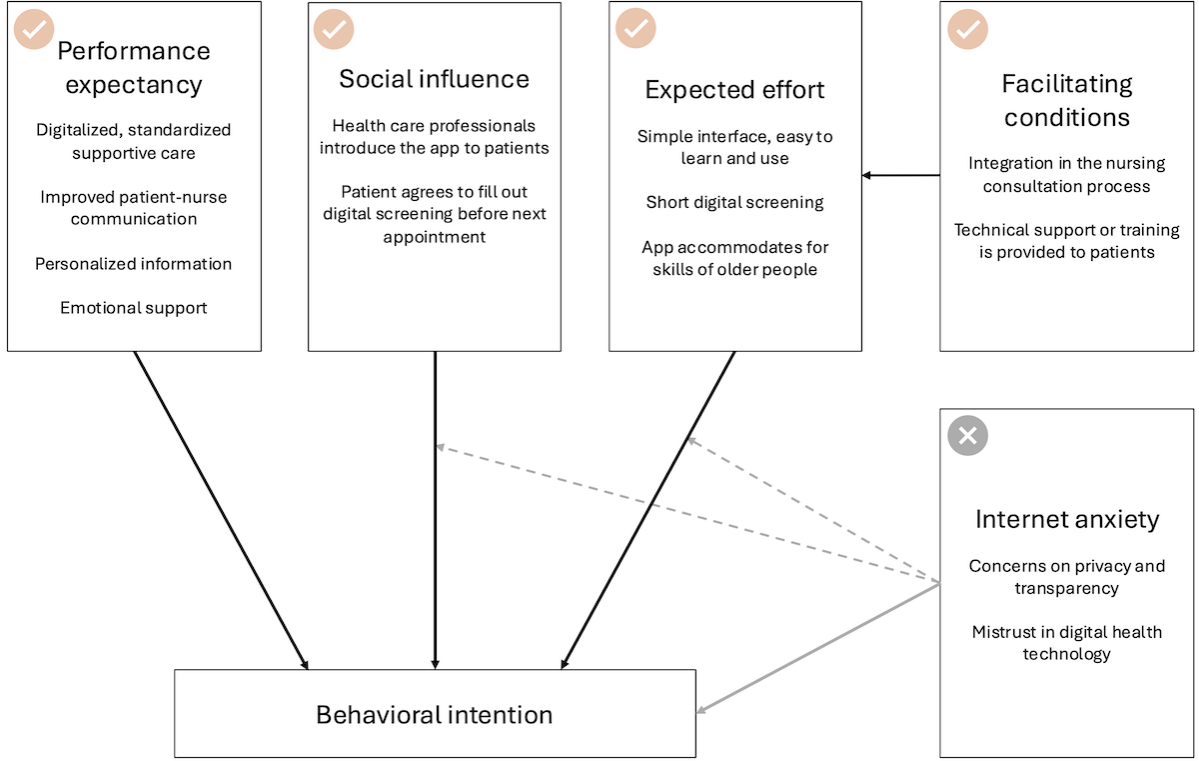
Unified Theory of Acceptance and Use of Technology model illustrating factors affecting acceptance of OncoSupport+. Performance expectancy, social influence, and expected efforts positively correlate behavioral intention (black lines), whereas internet anxiety negatively correlates with behavioral intention (gray line) and moderates the relationships between social influence and expected effort (gray dotted line).

Factors influencing the potential uptake of OncoSupport+ were mapped to the UTAUT framework (Figure 5; Multimedia Appendix 1 shows supporting quotes). Improved communication with health care professionals was the strongest driver of performance expectancy. Social influence was also important, as patients indicated they would be more likely to use the app if recommended during consultations. Effort expectancy was reflected in the preference for a simple, intuitive interface, particularly among older users or those with lower digital literacy. Facilitating conditions included integration into routine nursing consultations and the availability of technical support. Conversely, privacy concerns and internet anxiety emerged as barriers that may limit adoption.

### Prototyping

In the prototyping phase, the requirements identified in earlier stages were translated into user interface designs and iteratively refined with patient advocates and cancer nurses. A user flow was first created to map the patient and health care professional (ie, cancer nurses) journey through the app. Patients can self-register, whereas cancer nurse accounts, which allow access to multiple patient records, can only be created by the OncoSupport+ development team to ensure proper verification and compliance with data security protocols.

**Figure 6:**
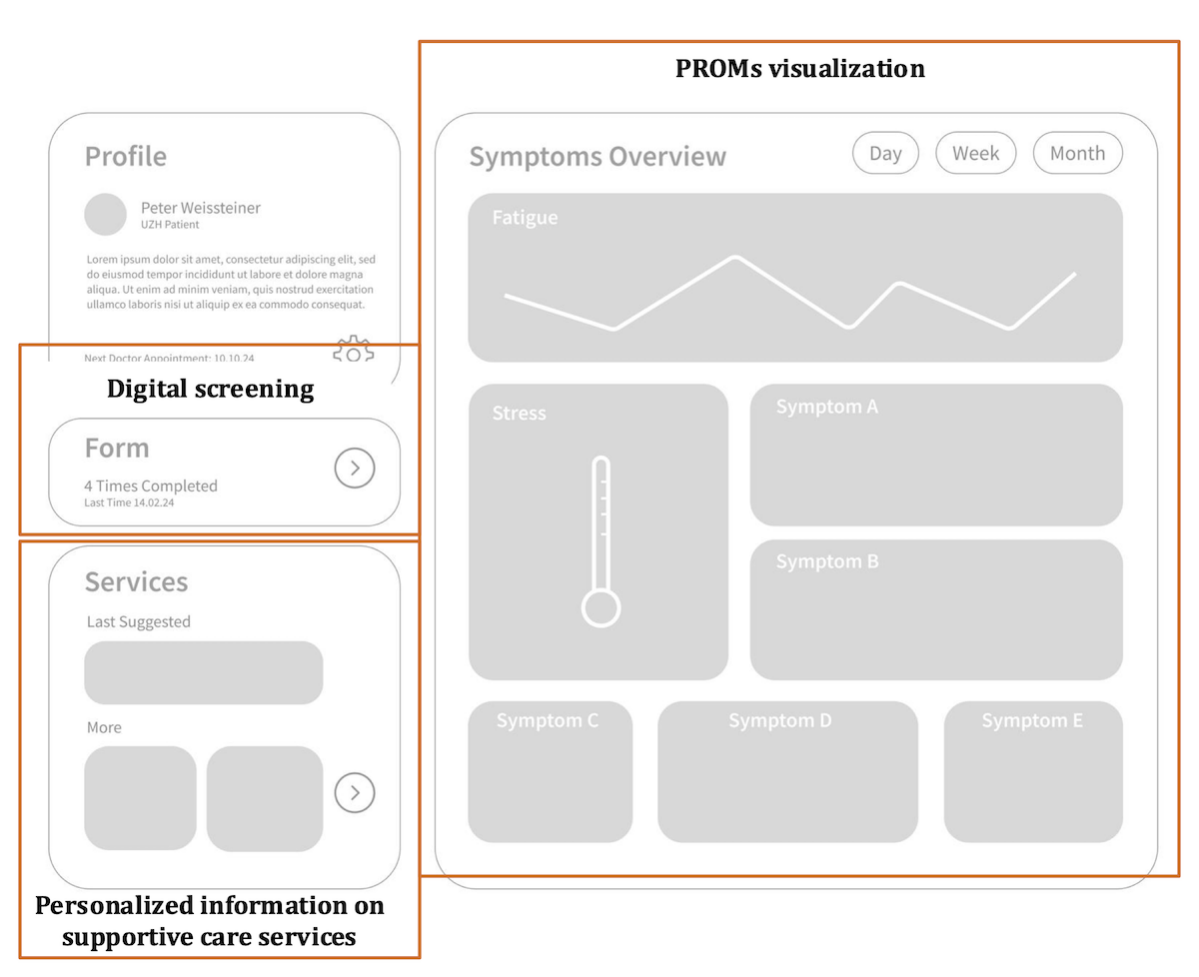
First version of the low-fidelity prototype displaying the main functionalities: digital screening, personalized information on supportive care services, and patient-reported outcome measures (PROMs) visualization.

The first low-fidelity prototype (Figure 6) underwent usability testing, where some patient advocates expressed discomfort with frequently viewing symptom trajectories. This led to relocating the symptom overview to a dedicated section, while the digital screening and personalized information sections were emphasized.

**Figure 7:**
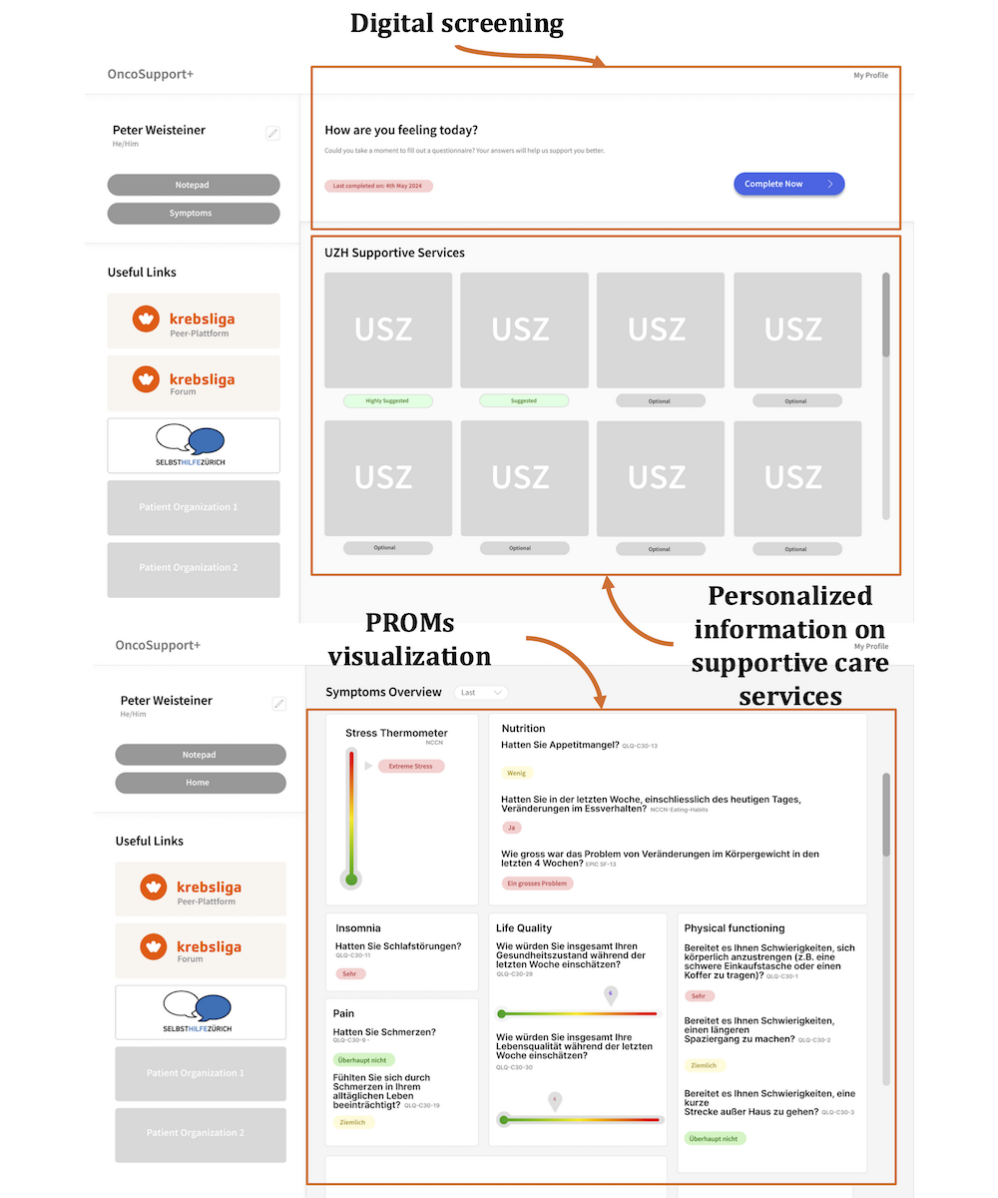
Second version of the low fidelity prototype displaying the main functionalities: digital screening, personalized information on supportive care services, and patient-reported outcome measures (PROMs) visualization.

A second low-fidelity prototype (Figure 7) underwent further feedback from patients and one cancer nurse. The cancer nurse recommended replacing red and green color schemes with neutral tones and adding bar charts to facilitate intuitive interpretation of symptom trends. Patient advocates suggested replacing clinical images with neutral, calming visuals and simplifying terminology.

**Figure 8:**
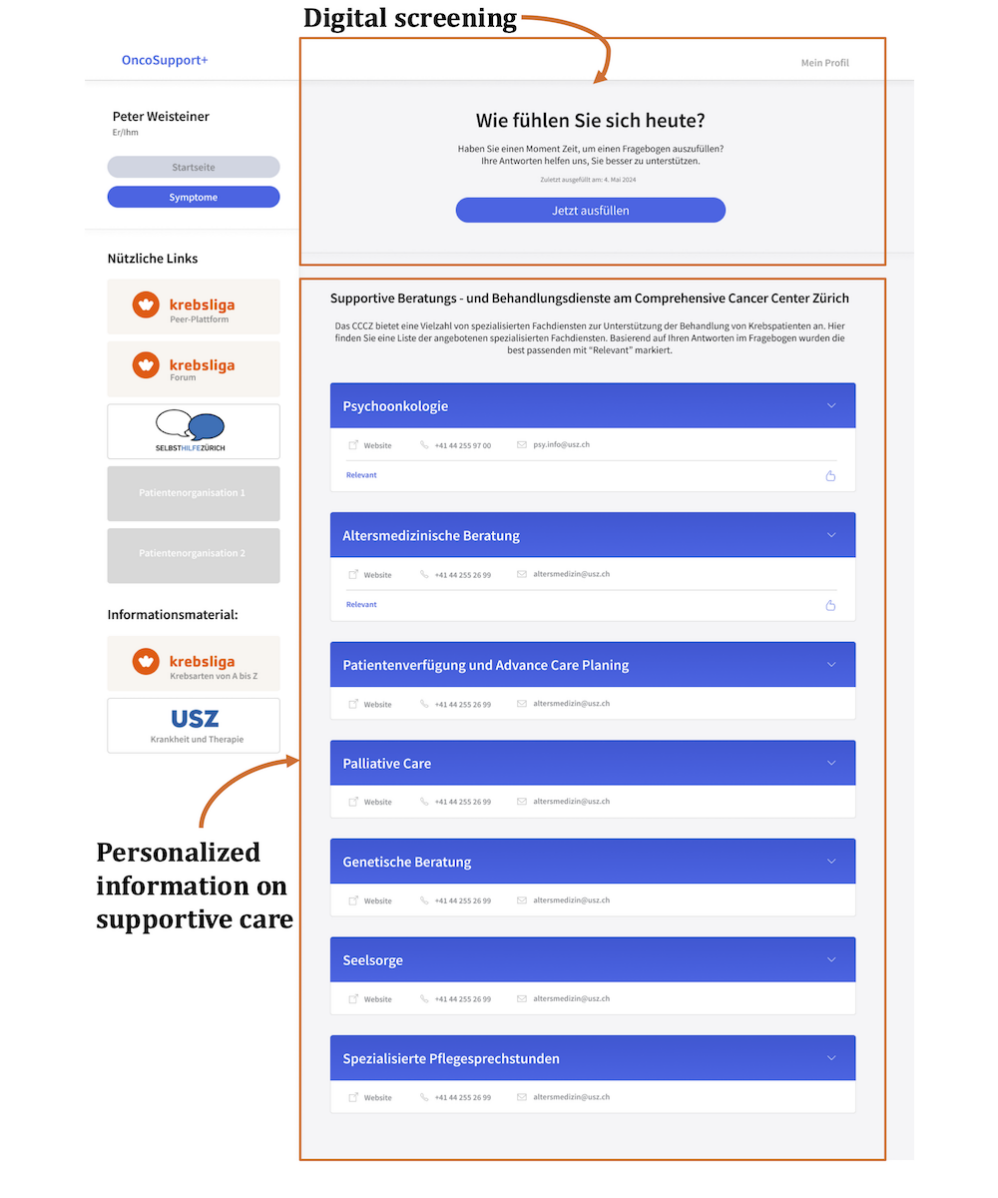
High-fidelity prototype showing the digital screening functionality and the personalized information on supportive care functionality.

Based on this feedback, a high-fidelity prototype (Figure 8) with responsive design was developed, compatible with both smartphones and laptops. A landing page was introduced to increase transparency about data storage and use and to highlight the key benefits of the app, particularly its potential to enhance communication between patients and health care professionals. Calming images were also integrated to improve the user experience. More details about the IT infrastructure can be found in Multimedia Appendix 4.

## Discussion

### Principal Results

This study reports the end-to-end co-design of OncoSupport+, a supportive cancer care app developed with patients with cancer and cancer nurses. The co-design approach identified challenges in supportive care and opportunities for digital health technology, generated a set of functionalities to address these challenges, and iteratively produced a prototype. As improving patient–health care professional communication and standardization and digitalization of supportive care were primary challenges, the OncoSupport+ app was designed to integrate a digital PROMs screening for supportive care using standardized oncology questionnaires (ie, EORTC QLQ-C30 and the NCCN Distress Thermometer) into the supportive care pathway. In addition, as enhancing self-efficacy, managing information overload, and receiving support from peers and health care professionals appeared to be very important, OncoSupport+ prepares patients for in-person nursing consultations by providing personalized information, access to supportive care services, and links to self-help groups, patient-to-patient chats, and stories from other patients with cancer. These features may prepare patients effectively for consultations, thereby improving the overall quality of the interaction between patients and health care professionals and their sense of empowerment. While meeting the performance expectations of patients and health care professionals is a primary driver for the uptake of the technology [28], the development of an easy-to-use app, its introduction by health care professionals, and the provision of technical support may be critical for improving overall adoption. However, concerns related to interacting with a digital environment (eg, internet anxiety) may hinder this adoption. Future usability and feasibility studies will further evaluate the implementation feasibility, real-world usability, and acceptance of OncoSupport+ in clinical practice.

### Comparison With Previous Work

The challenges identified in this study highlight the critical role of communication between patients and health care professionals, a domain where digital health technologies may play an important role. These technologies are not intended to replace health care professionals but rather to enhance and facilitate interactions with patients [32]. Consistent with previous research and systematic reviews [3], effective communication has been recognized as a key supportive care need. Not surprisingly, effective communication in cancer care has long been associated with improved medical and psychosocial outcomes, including reduced distress and increased satisfaction for both patients and clinicians [33]. Moreover, the European Society for Medical Oncology supports the integration of digital symptom monitoring, including the assessment of PROMs, as a vital element of cancer supportive care [34]. Evidence suggests that the use and digitalization of PROMs significantly enhance communication between patients and health care professionals [35]. For instance, a systematic review by Yang et al [35] suggests several mechanisms through which PROMs influence communication. These include increasing symptom awareness for both patients and health care professionals, prompting discussions (eg, addressing patient fears or uncertainty about disclosing symptoms), streamlining consultations (eg, using PROMs as a guide to direct health care professionals toward key patient-reported symptoms), and facilitating interprofessional communication between health care professionals (eg, enabling standardized patient tracking and optimizing the transfer of information). These mechanisms illustrate how PROMs may be a valuable tool for improving the quality of communication and, thereby, potentially enhancing clinical outcomes.

The findings of this study suggest that improving empowerment and self-efficacy in patients with cancer may be an important aspect of digital interventions in supportive care, consistent with previous research in this field. Recent systematic reviews [36,37] have indicated that digital health interventions, such as mobile apps and web-based platforms incorporating educational resources, interactive tools, medication reminders, and self-management features, may encourage patients to actively engage in their health care by enhancing empowerment and increasing self-efficacy. This enhanced sense of empowerment has been associated with better adherence to treatment regimens and more proactive health behaviors [37]. However, while digital health interventions that incorporate educational resources and self-management tools have been more consistently associated with increased self-efficacy, their direct impact on clinical outcomes, such as symptom reduction and improvements in quality of life, remains inconsistent across studies [38]. Although OncoSupport+ includes features related to informational and educational resources, appointment reminders, and tools to facilitate patient interaction, future research may explore the integration of self-management advice for symptom management to further enhance patient empowerment [39]. Additionally, while chat functions and telemedicine tools could further improve communication and self-efficacy, these features were not feasible for implementation in OncoSupport+. As patients value such tools, future research may focus on exploring the role of telemedicine solutions or artificial-intelligence–based chatbots in enhancing patient outcomes.

The digitalization of supportive care screenings and processes presents both significant opportunities and considerable challenges for hospitals. Digital transformation is reshaping health care by enhancing clinical workflows and promoting patient-centered care [40]. Implementing digital tools can improve efficiency and the quality of care, thereby reducing waste in health care costs. For instance, as reported by health care professionals interviewed in our participatory study, a separate study has shown that digital questionnaires significantly reduce the administrative burden on nurses compared to traditional paper-based questionnaires, allowing them to dedicate more time to patient care and resulting in substantial reductions in paper consumption [41]. Similarly, the adoption of electronic health record systems has been shown to facilitate better patient data management and streamline workflows. However, the transition to digital systems is not without challenges. Hospitals often face issues such as interoperability, strict data security regulations, the need for comprehensive staff training, and potential resistance to change among both health care professionals and patients [40]. This has been shown for cancer care as well [42]. Additionally, the lack of standardization across different IT systems at the hospital can hinder the seamless integration of digital tools. For example, the unsuccessful implementation of a nationwide digital patient dossier in Switzerland highlights the difficulties associated with a fragmented health care infrastructure and the absence of standardized frameworks [43]. Therefore, while digitalization offers promising benefits, careful consideration and strategic planning are essential to effectively navigate the associated challenges.

This study explored factors influencing the future acceptance of digital health apps like OncoSupport+, aligning with the UTAUT framework, which has been validated using quantitative data from digital health interventions [28]. An interesting finding concerns internet anxiety and the observation that age does not appear to be a barrier to technology acceptance. Internet anxiety, defined as the fear or discomfort individuals experience when using online technologies, can hinder the adoption of digital health solutions [28]. Previous research indicates that while age is not a significant predictor of health technology acceptance [44], it correlates with internet anxiety—an important factor in adoption. Moreover, individuals with lower digital literacy [45] tend to exhibit higher levels of internet anxiety. Although these results appear to be in line with previous literature, a larger and more representative sample may confirm the role of internet anxiety in relation to digital health technology acceptance in patients with cancer. However, providing adequate training and support can help alleviate these concerns, thereby enhancing the adoption of digital health technologies.

### Strengths and Limitations

This study has both strengths and limitations that should be considered when interpreting the results. The relatively small sample size (N=10 patients with cancer and patient advocates) may limit generalizability. However, the principle of thematic saturation was applied to ensure that no new themes emerged, supporting the adequacy of the sample. In addition, the inclusion of a significant number of female participants may account for gender-related biases in digital health acceptance. The patient group may not fully represent the broader population of patients with cancer, as most participants were aged below the average age of people with cancer and had a tertiary education level, higher than the Swiss national average. As education level correlates with digital literacy and some participants were highly experienced with digital health tools, acceptance of OncoSupport+ may differ in populations with lower digital experience. While individual interviews provided valuable insights, future research may benefit from workshops involving different user groups (eg, patients, nurses, family members, and caregivers) to foster mutual understanding and refine the co-design process. Although this study primarily examined user acceptance using the UTAUT framework, future implementation studies may apply frameworks such as Nonadoption, Abandonment, Scale-up, Spread, and Sustainability framework [46] or the Consolidated Framework for Implementation Research [47] to explore technology, organizational, and system-level factors affecting implementation and adoption. A key strength of this study is the direct integration of perspectives of patients, ensuring that OncoSupport+ is aligned with user needs and expectations, and the use of a structured co-design framework to guide development.

### Conclusion

This study highlights the feasibility and importance of collaborative design in developing digital health applications and interventions, emphasizing the need for direct involvement of patients and health care professionals in the development process. The findings suggest that digital interventions such as OncoSupport+ may include features and functionalities to enhance patient–health care professional communication, facilitate the digitalization of supportive care screenings, and support patient empowerment and self-efficacy by providing personalized information on supportive care services, promoting connection with other patients, and offering self-management advice. However, while OncoSupport+ appears to meet the performance expectations of both patients and health care professionals, future research is needed to evaluate its implementation feasibility and determine whether it can effectively improve communication, enhance self-efficacy, and ultimately lead to better health outcomes, such as improved quality of life.

## Appendix

Multimedia Appendix 1 Thematic analysis and results.

Multimedia Appendix 2 Digital screening questionnaires.

Multimedia Appendix 3 Knowledge-based algorithm to connect patient-reported outcome measures to supportive care services.

Multimedia Appendix 4 IT Infrastructure.

## Abbreviations

EORTC QLQ-C30: European Organization for Research and Treatment of Cancer Quality of Life Questionnaire Core 30
ePROM: electronic patient-reported outcome measure
mHealth: mobile health
NCCN: National Comprehensive Cancer Network
PROM: patient-reported outcome measure
SAKK: Swiss Group for Clinical Cancer Research
UTAUT: Unified Theory of Acceptance and Use of Technology

## Data Availability

The dataset generated and analyzed during this study is not publicly available because it is being used for the ongoing development of the digital health app OncoSupport+ at the University Hospital Zurich, but it is available from the corresponding author upon reasonable request.
