# Supplementary material for: Collaborative Design and Development of a Patient-Centered Digital Health App for Supportive Cancer Care: Participatory Study
Source: JMIR Hum Factors. 2025 Nov 11;12:e73829. doi: 10.2196/73829 (PMC12648126; doi:10.2196/73829)
Supplement: Multimedia Appendix 1 [file humanfactors_v12i1e73829_app1.docx]

# Multimedia Appendix 1: Thematic Analysis and Results

## Results: Themes and quotes from qualitative research

### Challenges in Cancer Supportive Care and Opportunities for Digital Health

**Theme 1: Need for Digitalization and Standardization of Supportive Care**

- "At the moment, we give patients a paper-and-pencil version of the screening when they come to the hospital. […] So, the digital screening should actually improve this by allowing patients to answer in advance […]. It should also improve the screening rate and relieve nurses from having to conduct the screening themselves. This way, patients can complete it at home, and we can spend more time discussing their responses during the consultation." (Cancer Nurse 2, focus group)
- “We have to look for our patients ourselves and at the moment [to do a nursing consultation]. We try to meet those patients who receive their first [chemo or immuno] therapy. This takes a lot of time, so it’s not very efficient.” (Cancer Nurse 1, focus group)
- "I think in the beginning, it’s helpful to have, you know, the links [to supportive care services]." (Patient advocate 2)
- "And first, I didn’t think about nutrition counseling or exercise. . . the medical treatment was in the focus. But later I realized I had to check my weight more carefully and try to do sports again." (Cancer Patient 4)

**Theme 2: Communication - Highly valued but Challenging**

- "It's difficult [the communication with healthcare professionals]... most of the time, my oncologist answers quite quickly. But sometimes he has too many things to do."(Patient advocate 3)
- "Sometimes I have symptoms, and I don’t know if I need to see a doctor. It would be nice to have someone to ask." (Cancer Patient 6)
- "I would love to have a reference nurse that answers within 48 hours. Even if I have to call several times, I need to know I can get an answer." (Patient advocate 1)
- " When we start therapy, we give them medication that makes them very tired and then they [the patients] don’t want to complete the paper-based questionnaire." (Cancer nurse 1, focus group)
- “I think one of the big problems is that we have a lot of foreign language speaking patients.” (Cancer nurse2, focus group)

**Theme 3: Improving Self-Efficacy and Managing Information Overload**

- "Sometimes you forget to ask about these services, so having them listed in the app would be useful." (Cancer Patient 3)
- "I get a lot of information from different sources, but it would be easier if there was a single, reliable place where I could find everything I need."(Patient advocate 2)
- "Having an app where I can keep track of my symptoms and needs would help me remember what to discuss with my doctor." (Cancer Patient 3)
- "If I knew what to expect, I would feel more in control. It helps when I can prepare questions in advance." (Cancer Patient 5)
- "I have read a few things but, in the end, I was so overwhelmed with the conversations. Before each surgery, you have a conversation with the surgeon or the radiologist and then with the anesthetist and then again, a conversation and then you get a few more documents […]" (Cancer Patient 6)
- “But it is difficult to tell which resources are trustworthy, where can I get informed?” (Cancer Patient 1)

**Theme 4: Need for Connection, Medical and Social Support**

- “If I had an app for my illness, it would be good, but it would make everything so virtual, and you still need to connect with people in real life.” (Cancer Patient 4)
- “And also, I think that if you can talk a little bit with people your own age [this would be good]. It doesn’t have as many young patients [on the peer platform]. It would be good if you could talk to people in the same age group. I don’t mind sharing with older patients, not at all, but sometimes it’s just different with people the same age.” (Cancer Patient 5)
- “But to talk to people who understand you. Were you can say “I’m really tired today I really just made dinner, and I didn’t do the dishes” where people also say “Yes, I know what you are talking about, sometimes I’m very tired”. That’s certainly a good thing, I think.” (Cancer Patient 3)
- “I went to the patient organization X to have information, to also have a second opinion and because I found people who gave me comfort.” (Patient advocate 3)
- “For me it does not give me energy to share with people in similar situation how my

prognosis is. I know there are people whom self-help groups can help. For me I don’t look for that, and it does not help me to exchange with others about my cancer journey.” (Cancer Patient 2)

- “I don’t know how it’s for you, but there are so many questionnaires about so many different things. And so, after a while, I prefer to talk with someone.” (Patient advocate 2)

### Functionalities Proposed by Patients

Below are quotes from patients and advocates supporting the functionalities suggested by at least five participants.

**Patient-Healthcare Professional Chat**

- “Yeah, but what I’m thinking now, it could be helpful that a nurse would answer questions over the app you know, so that you can have a little chat.” (Patient advocate 2)
- “A video communication or a chat, that can certainly help. In my opinion, it would

not have to be a doctor. If everyone understands that, then the first questions can be answered.” (Patient advocate 3)

- “Or if there is such a chat where you can reach someone.” (Cancer Patient 5)
- “That’s why I said, if you have an app where you can ask specific questions, […], such a chat would be very easy.” (Cancer Patient 6)
- “Yes, this [chat with nurses] would be good for medical questions, when the doctors are busy.” (Cancer Patient 6)

**Patient-Patient Chat**

- “And that there may also be a function where you can contact other patients.” (Patient advocate 2)
- “If I have possible contacts to other people and it is displayed like “these people are open for a conversation”, this I would really be welcoming.” (Patient advocate 2)
- “I can also look for individual people who might be very detailed: different types of cancer for example. I can click on them, then I see each type of cancer. There are maybe two or three people who make themselves available as a peer or something, where I can make a call and maybe also write an e-mail.” (Patient advocate 3)
- “Yes, it’s interesting if you could also exchange with other people, whom the same

things are happening.” (Cancer Patient 4)

- “And I also think that if you can talk a little bit with people your own age [this would be good]. It doesn’t have as many young patients [on the peer platform of Krebsliga]. It would be good if you could talk to people in the same age group. I don’t mind sharing with older patients, not at all, but sometimes it’s just different with people the same age.” (Cancer Patient 5)
- “Or maybe you can exchange with someone. Or you sit in the waiting room, and than you can ask people if they are also in the app. You could say: we can text sometime.” (Cancer Patient 4)

**Check Symptoms and Side effects**

- "And maybe, I don’t know exactly what you are developing, but if it also allows you to enter symptoms or track how you feel every day, I think it would enable very quick communication between the patient and the hospital." (Cancer patient 4)
- "I think an app that helps determine whether the symptoms you have are very severe or not too bad would be helpful." (Patient advocate 1)
- "If I can easily see what side effects might occur or compare my symptoms, that would be useful. It would also help me understand how to cope with my illness and diagnosis." (Patient advocate 3)
- "I had nausea after the first day and night following my first treatment, and it went away after three days… Is that something included in the app?" (Cancer patient 1)

**Self-management advice**

- “For example, if my symptoms are severe, I would want clear step-by-step guidance, and ideally, the process should be quick.” (Patient advocate 1)
- “If there is such an app, then it is easier to search for nausea and then the solution comes right away.” (Cancer Patient 5)
- “What can I do if this case occurs? Or what can I do if I have anxiety, for example, or if I can’t sleep or if I have to vomit a lot?” (Patient advocate 2)

### Potential Factors impacting on the future acceptance of OncoSupport+

### **Performance Expectancy**

Performance expectacy quotes align with the section Challenges in Cancer Supportive Care and Opportunities for Digital Health.

### **Social Influence**

Patients indicated that encouragement from healthcare professionals could motivate them to engage with the app and complete digital screenings.

- "Maybe if I would agree with you the next time I come here I have filled it out then I would put it away first, but maybe the moment comes when I’m like 'Ohh I’ll go to Zurich and I’ll meet Ms. XXX again who told me to fill it out.'" (Cancer Patient 1)
- "And so it is often the medical information [from a doctor] that they consider to be more important for themselves, or their understanding of it." (Cancer nurse 2, focus group)

### **Effort Expectancy**

Patients emphasized the need for a simple, intuitive app, particularly for older users, and highlighted that lengthy questionnaires could discourage engagement.

- "If it is complicated to use, I don’t think people will use it, especially older patients. It has to be really intuitive." (Cancer Patient 5)
- “Well, what is important is that the use is as simple as possible.” (Patient advocate 3)
- “I participated in a study where I had to fill in a questionnaire, and there were 200 questions. I think the patient doesn’t want to answer so many questions.” (Patient advocate 2)
- “If it is a lot of questions coming up, I ... I am the person who does not do it.” (Cancer Patient 6)

### **Internet Anxiety**

Some patients expressed concerns regarding privacy, transparency, and trust in digital health technology and being afraid to loose the contact with the healthcare professional.

- “But I have, yeah, for me, it's difficult to give private data on an app, any kind of... Okay, I just don't want everybody to know that I was feeling bad that day.” (Cancer Patient 5)
- “I hate that. I mean, I have the feeling to give intimate things to the cloud. Okay. So, it’s a privacy problem. Yeah. There is the question of privacy, and I don’t see what it could bring.” (Patient advocate 1)
- “ […] You will lose human contact, that's it. Otherwise, you can be, as they say, reachable, day and night, you can communicate with someone, day and night, there are others in the opposite direction of the medal”. (Patient advocate 2)
