# Supplementary material for: Collaborative Design and Development of a Patient-Centered Digital Health App for Supportive Cancer Care: Participatory Study
Source: JMIR Hum Factors. 2025 Nov 11;12:e73829. doi: 10.2196/73829 (PMC12648126; doi:10.2196/73829)
Supplement: Multimedia Appendix 2 [file humanfactors_v12i1e73829_app2.docx]

# Multimedia Appendix 2: Digital Screening questionnaires

OncoSupport+ incorporates digital questionnaires from validated Patient-Reported Outcome Measures (e.g., EORTC QLQ-C30, Distress Thermometer, and selected items from IPOS 3 and EPIC SF-13), along with preference, onboarding, and clinical questions. These assessments are used during nursing consultations and help enhance the personalization of information within the OncoSupport+ app.

## 1. Patient-Reported Outcomes Measures

| QLQ-C30-1 | Do you have any trouble doing strenuous activities, like carrying a heavy shopping bag or a suitcase? | Not at all (1), A little (2), Quite a bit (3), Very much (4) |
| --- | --- | --- |
| QLQ-C30-2 | Do you have any trouble taking a long walk? | Not at all (1), A little (2), Quite a bit (3), Very much (4) |
| QLQ-C30-3 | Do you have any trouble taking a short walk outside of the house? | Not at all (1), A little (2), Quite a bit (3), Very much (4) |
| QLQ-C30-4 | Do you need to stay in bed or a chair during the day? | Not at all (1), A little (2), Quite a bit (3), Very much (4) |
| QLQ-C30-5 | Do you need help with eating, dressing, washing yourself or using the toilet? | Not at all (1), A little (2), Quite a bit (3), Very much (4) |
| QLQ-C30-6 | Were you limited in doing either your work or other daily activities? | Not at all (1), A little (2), Quite a bit (3), Very much (4) |
| QLQ-C30-7 | Were you limited in pursuing your hobbies or other leisure time activities? | Not at all (1), A little (2), Quite a bit (3), Very much (4) |
| QLQ-C30-8 | Were you short of breath? | Not at all (1), A little (2), Quite a bit (3), Very much (4) |
| QLQ-C30-9 | Have you had pain? | Not at all (1), A little (2), Quite a bit (3), Very much (4) |
| QLQ-C30-10 | Did you need to rest? | Not at all (1), A little (2), Quite a bit (3), Very much (4) |
| QLQ-C30-11 | Have you had trouble sleeping? | Not at all (1), A little (2), Quite a bit (3), Very much (4) |
| QLQ-C30-12 | Have you felt weak? | Not at all (1), A little (2), Quite a bit (3), Very much (4) |
| QLQ-C30-13 | Have you lacked appetite? | Not at all (1), A little (2), Quite a bit (3), Very much (4) |
| QLQ-C30-14 | Have you felt nauseated? | Not at all (1), A little (2), Quite a bit (3), Very much (4) |
| QLQ-C30-15 | Have you vomited? | Not at all (1), A little (2), Quite a bit (3), Very much (4) |
| QLQ-C30-16 | Have you been constipated? | Not at all (1), A little (2), Quite a bit (3), Very much (4) |
| QLQ-C30-17 | Have you had diarrhea? | Not at all (1), A little (2), Quite a bit (3), Very much (4) |
| QLQ-C30-18 | Were you tired? | Not at all (1), A little (2), Quite a bit (3), Very much (4) |
| QLQ-C30-19 | Did pain interfere with your daily activities? | Not at all (1), A little (2), Quite a bit (3), Very much (4) |
| QLQ-C30-20 | Have you had difficulty in concentrating on things, like reading a newspaper or watching television? | Not at all (1), A little (2), Quite a bit (3), Very much (4) |
| QLQ-C30-21 | Did you feel tense? | Not at all (1), A little (2), Quite a bit (3), Very much (4) |
| QLQ-C30-22 | Did you worry? | Not at all (1), A little (2), Quite a bit (3), Very much (4) |
| QLQ-C30-23 | Did you feel irritable? | Not at all (1), A little (2), Quite a bit (3), Very much (4) |
| QLQ-C30-24 | Did you feel depressed? | Not at all (1), A little (2), Quite a bit (3), Very much (4) |
| QLQ-C30-25 | Have you had difficulty remembering things? | Not at all (1), A little (2), Quite a bit (3), Very much (4) |
| QLQ-C30-26 | Has your physical condition or medical treatment interfered with your family life? | Not at all (1), A little (2), Quite a bit (3), Very much (4) |
| QLQ-C30-27 | Has your physical condition or medical treatment interfered with your social activities? | Not at all (1), A little (2), Quite a bit (3), Very much (4) |
| QLQ-C30-28 | Has your physical condition or medical treatment caused you financial difficulties? | Not at all (1), A little (2), Quite a bit (3), Very much (4) |
| QLQ-C30-29 | How would you rate your overall health during the past week? | 1 (very poor), 2, 3, 4, 5, 6, 7 (excellent) |
| QLQ-C30-30 | How would you rate your overall quality of life during the past week? | 1 (very poor), 2, 3, 4, 5, 6, 7 (excellent) |
| NCCN-Thermometer | Distress is an unpleasant experience of a mental, physical, social, or spiritual nature. It can affect the way you think, feel, or act. Distress may make it harder to cope with having cancer, its symptoms, or its treatment.  Please circle the number (0–10) that best  describes how much distress you have been experiencing in the past week, including today. | 0 (No distress), 1, 2, 3, 4, 5, 6, 7, 8, 9, 10 (Extreme distress) |
| NCCN-Eating-Habits | Have you had concerns about changes in eating in the past week, including today? | Yes, no |
| IPOS 3 F3 | In the past 3 days, were you worried or concerned about your illness or treatment? | Not at all (0) Rarely (1) Sometimes (2) Mostly (3) Always (4) |
| IPOS 3 F4 | In the past 3 days, were your family or friends worried or concerned about you? | Not at all (0) Rarely (1) Sometimes (2) Mostly (3) Always (4) |
| IPOS 3 F6 | In the past 3 days, were you at peace with yourself? | Not at all (0) Rarely (1) Sometimes (2) Mostly (3) Always (4) |
| IPOS F9 | Were practical problems related to your illness (e.g., financial or personal issues) addressed? | No problems (0)  Problems mostly addressed (1)  Problems partially addressed (2)  Problems hardly addressed (3)  Problems not addressed (4) |
| EPIC SF-*13* | How big of a problem were changes in body weight in the last 4 weeks? | No problem (0)  Very small problem (1)  A small problem (2)  A moderate problem (3)  A big problem (4) |

## 2. Preference Questions

Preference questions are non-clinical questions that are used for providing personalized information about services. These questions are asked together with PROMs.

| P1 | Would you like to discuss your personal preferences for your treatment to ensure that we follow your wishes in case of a medical crisis or emergency? | Yes, No |
| --- | --- | --- |
| P2 | Do you need assistance in creating or updating an advance healthcare directive tailored to your preferences? | Yes, No |
| P3 | Besides your physical and mental health, are there other aspects (e.g., spiritual or religious beliefs) that you consider important for ensuring the best possible care? | Yes, No |
| P4 | Are you interested in a free beauty workshop designed to boost self-esteem and confidence in cancer patients? | Yes, No |

## 3. Onboarding Questions

Onboarding questions are questions asked during the registration in the app.

| O1 | Which age group do you belong to? | - 18-33 - 34-44 - 45 - 49 - 50 - 54 - 55 - 59 - 60 - 64 - 65-69 - 70-74 - 75-79 - 80-84 - 85-89 - 90-94 - 95 years and older |
| --- | --- | --- |
| O2 | What was your assigned sex at birth? | Female / Male |
| O3 | Do you smoke? | Yes, No |
| O4 | Have you had breast, ovarian, or colorectal cancer at a young age? | Yes, No |
| O5 | Do multiple members of your family have cancer? | Yes, No |
| O6 | Are you currently employed? | Yes, No |

## 4. Clinical questions

Clinical questions are related to clinical characteristics of the patient.

| C1 | Do you have advanced-stage cancer? | Yes, No |
| --- | --- | --- |
| C2 | What type of cancer has been diagnosed? | 1. Brain/Central Nervous System (CNS) 2. Lymphoma 3. Leukemia 4. Melanoma 5. Colorectal 6. Other gastrointestinal cancers 7. Gynecological (breast, ovarian, uterine) 8. Prostate/Testicular 9. Urinary system 10. Head and neck 11. Lung 12. Bone and soft tissue |
