# Supplementary material for: Collaborative Design and Development of a Patient-Centered Digital Health App for Supportive Cancer Care: Participatory Study
Source: JMIR Hum Factors. 2025 Nov 11;12:e73829. doi: 10.2196/73829 (PMC12648126; doi:10.2196/73829)
Supplement: Multimedia Appendix 3 [file humanfactors_v12i1e73829_app3.docx]

# Multimedia Appendix 3: Knowledge-based algorithm to connect Patient-Reported Outcomes Measures to Supportive Care Services

Table B1: Knowledge based algorithm to sort supportive care information and services based on medical knowledge

| **Service / Information** | **Patient Reported Outcome Measures and other questions** | **Rule, reference and rational** |
| --- | --- | --- |
| **Safety**  **Safety Information**  This app is not intended to be used as a basis for any diagnosis or treatment decisions; instead, it offers information on available supportive care services and related information. Your symptoms will be shared during nursing consultations. For immediate help or support, please contact your oncology treatment team directly. |  |  |
| **Psycho-oncology**  ***Information A***  Psycho-oncology offers psychiatric and psychotherapeutic treatment and support to patients with cancer and their relatives. The aim is psychological stabilization and improvement in the quality of life.  We offer support in dealing with stressful situations and coping with physical changes in individual consultations at the USZ Campus (Rämistrasse) or at the USZ Flughafen (The Circle). | **Distress Thermometer**  Please circle the number (0-10) that best describes how much stress you have experienced in the past week, including today.  **QLQ C-30 (Rating from 1 = ‘Not at all’ to 4 = ‘Very much’)**  *Emotional Functioning*  21. Did you feel tense?  22. Did you worry?  23. Did you feel irritable?  24. Did you feel depressed?  *Social Functioning*  26. Has your physical condition or medical treatment interfered with your family life?  27. Has your physical condition or medical treatment interfered with your social activities? | ***Rule A***  IF Distress Thermometer ≥ 5  OR  At least one of the QLQ C-30 Emotional Functioning questions (21, 22, 23, 24) and Social Functioning questions (26, 27) equals 4 (i.e., very much).  OR  At least one of the QLQ C-30 Emotional Functioning questions (21, 22, 23, 24) and Social Functioning questions (26, 27) increase two scale units compared to the previous filled-in PROMs.  THEN → ***Information A***  **Reference and rational**  According to the European Society for Medical Oncology (ESMO) guidelines ^1^, the Distress Thermometer, developed by the National Comprehensive Cancer Network, is recommended for routine use to identify clinically significant distress. A suggested threshold score of 5 or higher on this instrument may serve as an indicative marker for potential psychological and psychiatric disorders in oncology patients ^2^. The *emotional* and *social functioning* domains of the Quality-of-Life Questionnaire-Core 30 (QLQ-C30) are associated with unmet needs in the mental health of oncology patients. However, it is important to note that these tools alone cannot be used for diagnosing clinical disorders such as anxiety or depression in patients with cancer. Validated screening tools are necessary to assess the psycho-oncological needs of patients ^1^. |
| **Geriatric Medicine**  ***Information B***  We provide comprehensive advisory services for older patients with cancer. In addition to addressing physical functioning, our focus extends to areas such as nutrition, exercise, psychological factors, and the social environment. Depending on the individual's initial situation, we emphasize preventive and secondary preventive measures, including precautions and early detection. We also explore risk factors leading up to oncological treatment. Our goal is to collaboratively develop a personalized plan with recommendations and tips that you, as a patient, can easily incorporate into your everyday life. | **Onboarding question**  O1. What is your year of birth?  **QLQ C-30 (Rating from 1 = ‘Not at all’ to 4 = ‘Very much’)**  *Physical Functioning*  1. Do you have any trouble doing strenuous activities, like carrying a heavy shopping bag or a suitcase?  2. Do you have any trouble taking a long walk?  3. Do you have any trouble taking a short walk outside of the house?  4. Do you need to stay in bed or a chair during the day?  5. Do you need help with eating, dressing, washing yourself or using the toilet? | ***Rule B***  IF Age ≥ 70  AND  {At least one of the QLQ C-30 Physical Functioning questions (1, 2, 3, 4, 5) equals to 4 (i.e., very much)  OR  At least one of the QLQ C-30 Physical Functioning questions (1, 2, 3, 4, 5) increases two scale units compared to the previous filled-in PROMs}.  THEN → ***Information B***  **Reference and rational**  As per the guidelines established by the International Society of Geriatric Oncology, it is recommended for healthcare professionals engaged in cancer care to assess the Quality of Life (QoL) of older individuals aged 70 and above who are living with cancer. This assessment should be integrated into the decision-making process to shape the most effective cancer management strategies. Employing tools such as the EORTC QLQ-C30, with a particular focus on *functional* and *social* domains, can provide valuable insights into unmet needs within the geriatric population to consider during geriatric assessment ^3^. |
| **Living Will and Advance Care Planning**  *Information C*  We offer all patients a consultation during which we discuss potential future health crises related to their illness, including their treatment expectations and the limits of what can be addressed. This collaborative, forward-looking planning is known as Advance Care Planning (ACP). In these sessions, commonly referred to as ACP, you can share your expectations and concerns about potential medical treatments with a qualified ACP counselor.  Additionally, if you desire, we can assist you in documenting your expectations clearly and comprehensively through a 'plus' living will. This document not only summarizes your decisions concerning medical treatments but also includes emergency planning aspects. | **Preference question**  P3. We are here for you if:   - You would like to discuss your personal wishes and preferences for your treatment so that we can treat you according to your wishes in possible health crises and emergencies. - You would like support in drawing up a living will that is medically feasible and tailored to your wishes – or in updating your existing living will. | *Rule C*  IF At least one of the options in the preference question P3 is checked.  THEN → ***Information C*** |
| **Palliative care**  ***Information D***  We offer specialized palliative care to patients with acute symptoms that are difficult to treat, an unstable illness or other complex problems. In addition to the care on our  ward, we regularly offer palliative care consultations for outpatients. The focus of the advice is on the comprehensive and efficient treatment of cancer symptoms – regardless of whether the tumor is currently being treated or not. | **Distress Thermometer**  Please circle the number (0-10) that best describes how much stress you have experienced in the past week, including today.  **History of Cancer and Family Cancer History** [to ask during onboarding]  H3. Have been diagnosed with advanced cancer?   - Yes - No | ***Rule D***  IF History of Cancer and Family Cancer History question H3 is checked with ‘Yes’ (i.e., advanced cancer).  AND  Distress Thermometer ≥ 5  THEN 🡪 ***Information D***  **Reference and rational**  The identification of patients with potential unmet palliative care needs is often conducted by initially identifying those with advanced progressive diseases ^4^ (e.g., cancer) and subsequently assessing their unmet needs. One method for evaluating these needs is by using the Distress Thermometer; a score above 5 often indicates unmet needs in various domains, including physical, emotional, spiritual, or informational. |
| **Genetic Counseling**  ***Information E***  Genetic counseling can be beneficial in cases where there is a high incidence of cancer in a family or if it occurs at a young age, especially in the case of breast, ovarian, and intestinal tumors. The primary purpose of genetic counseling is to document and assess facts from the family and personal history of the disease.  We provide information to those seeking advice about their results, including the risk of cancer, the possibilities of genetic testing, and the preventive measures that arise from this. Additionally, we offer counseling for the children of patients who may be affected by hereditary forms of cancer. | **History of Cancer and Family Cancer History** [to ask during onboarding]  H1. Have you had at a young age breast, ovary or colon cancer?   - Yes - No   H2. Are there several people in your family with cancer?   - Yes - No | ***Rule E***  IF History of Cancer and Family Cancer History question H1 OR H2 is checked with ‘Ja’.  THEN → ***Information E***  **Reference and rational**  Specific features in a person's medical or family history (e.g., early age at cancer diagnosis and multiple generations affected by tumors or cancer) may indicate a hereditary syndrome, prompting the need for more detailed genetic screening in patients with cancer ^56^. |
| **Exercise/Sport program**  ***Information F***  At CCCZ, an interdisciplinary team comprising experts in sports medicine, physiotherapy, occupational therapy, sports therapy, sports science, and oncology collaborates closely. This team, drawn from the University Hospital Zurich and the University Hospital Balgrist, is dedicated to providing cancer patients with personalized training programs. These programs are tailored to individual needs, guided by professionals, and based on the latest scientific findings.  Our services include:   - Screening and needs analysis - Individualized advice and training management - Personalized multimodal exercise, incorporating physiotherapy and occupational therapy (endurance, strength, sensorimotor, cognitive, and vibration training, as well as yoga/pilates) - Sport-specific training - Performance diagnostic tests for training control | **QLQ C-30 (Rating from 1 = ‘Not at all’ to 4 = ‘Very much’)**  *Physical Functioning*  1. Do you have any trouble doing strenuous activities, like carrying a heavy shopping bag or a suitcase?  2. Do you have any trouble taking a long walk?  3. Do you have any trouble taking a short walk outside of the house?  4. Do you need to stay in bed or a chair during the day?  5. Do you need help with eating, dressing, washing yourself or using the toilet?  *Fatigue*  During the past week:  10. Did you need to rest?  12. Have you felt weak?  18. Were you tired? | ***Rule F***  IF  {At least one of the QLQ C-30 Physical Functioning questions (1, 2, 3, 4, 5) or Fatigue questions (10, 12, 18) equals to 4 (i.e., very much)  *OR*  At least one of the QLQ C-30 Physical Functioning questions (1, 2, 3, 4, 5) or Fatigue questions (10, 12, 18) increase two scale units compared to the previous filled-in PROMs}.  THEN → ***Information F***  **Reference and rational**  Fatigue and impaired physical functioning may be indicative of cancer-related fatigue for which exercise and sport programs may be highly recommended ^7^. |
| **Nutrition Counseling**  ***Information G***  We provide support for all questions related to nutrition and guide you step by step in making dietary changes in your everyday life. Cancer diseases and therapies can lead to issues such as weight loss, loss of appetite, nausea, digestive problems, or changes in taste. Additionally, many patients wonder about the best type of diet to support cancer therapy. | **QLQ C-30 (Rating from 1 = ‘Not at all’ to 4 = ‘Very much’)**  *Appetite loss*  During the past week:  13. Have you lacked appetite?  **Changes in eating habits and weight**  E1. In the past week, including today, have you had any complaints about changes in eating habits?   - Yes - No   E2. How big of a problem has changes in body weight been in the past 4 weeks?   - Not a problem - Very small problem - A small problem - A moderate problem - A big problem | ***Rule G***  IF EORTC QLE-C30 Appetite Loss question 13 equals to or greater than ‘A little bit’  OR  Changes in eating habits and weight questions E1 equals to ‘Yes’.  OR  Changes in eating habits and weight questions E2 equals to or greater than ‘A small problem’.  THEN → ***Information G***.  **Reference and rational**  Cancer, tumor-induced metabolic changes (often referred to as cancer-induced malnutrition or cachexia), and antineoplastic treatments can cause appetite loss, changes in eating habits, and weight fluctuations ^8,9^. Nutritional Risk Screening may be necessary to identify the need for nutritional advice. |
| **Smoking advice**  ***Information H***  Tobacco use increases the risk of cancer and vascular diseases. Additionally, tobacco use can cause non-cancerous respiratory diseases, infections, osteoporosis, tooth and gum disease, and impaired senses of taste, smell, and vision. This advice is targeted at individuals who wish to quit smoking.  Smoking consultations are available on Wednesdays and Fridays at the Cardiology Clinic. | **Onboarding question**  O3. Do you smoke?   - Yes - No | ***Rule H***  IF smoking = yes  THEN **→ *Information H***  **Reference and rational**  As tobacco use not only causes cancer but also negatively impacts cancer treatment and survival, ESMO highlights the role of oncologists in routinely identifying their patients who use tobacco and supporting them to quit ^10^. |
| **Social services**  ***Information I***  An illness, an accident, or pregnancy can raise many questions. Patients are suddenly confronted with hospital treatment, a changed state of health, or even a new life situation. Serious illnesses often necessitate rethinking and restructuring one's life.  At the social counseling service, we provide advice on financial burdens, questions about social security law, optimal follow-up care, and psychosocial issues. Additionally, we connect you with counseling centers and institutions outside of Zurich University Hospital.  Your doctor or a nurse at the USZ can register you for our social counseling service. You or your relatives are also welcome to contact us. | **QLQ C-30 (Rating from 1 = ‘Not at all’ to 4 = ‘Very much’)**  *Social Functioning*  26. Has your physical condition or medical treatment interfered with your family life?  27. Has your physical condition or medical treatment interfered with your social activities?  *Financial difficulties*  28. Has your physical condition or medical treatment caused you financial difficulties?  **IPOS 7**  *Practical problems*  F9. Have any practical problems arising from your illness been addressed (e.g., financial or personal)?   1. No problems to address. 2. Problems mostly addressed. 3. Problems partially addressed. 4. Problems barely addressed. 5. Problems not addressed. | ***Rule I***  IF  At least one of the QLQ C-30 Social Functioning questions (26, 27) and Financial difficulties (28) equals 4 (i.e., very much).  *OR*  At least one of the QLQ C-30 Social Functioning questions (26, 27) and Financial difficulties (28) increase two scale units compared to the previous filled-in PROMs.  *OR*  IPOS 7 question Practical Problems F9 equals 3 (i.e., ‘Problems barely addressed’, ‘Probleme kaum angegangen’) OR 4 (i.e., ‘Problems not addressed’, ‘Probleme nicht angegangen’)  *OR*  IPOS 7 question Practical Problems F9 increase of two scale units compared to the previous filled-in PROMs.  THEN → ***Information I*** |
| **Pastoral Care**  *Information L*  During a hospital stay, some people would like a sympathetic ear or support on their journey, while others draw strength and confidence from their religious tradition in times of illness or change. The hospital chaplaincy team is here to support you with your concerns. | **QLQ C-30 (Rating from 1 = ‘Not at all’ to 4 = ‘Very much’)**  22. Did you worry?  **IPOS 7**  F6. Were you at peace with yourself?   - Always - Mostly - Sometimes - Rarely - Not at all   **Preference question**  P6. In addition to your physical and mental health, is there anything else, such as your spiritual or religious beliefs, that you think is important to provide the best care for you?   - Yes - No | *Rule L*  IF Preference question P6 equals ‘Ja’.  *AND*  {QLQ C-30 question 22 equals to ‘Sehr’  *OR*  QLQ C-30 question 22 increases of two scale units compared to the previous filled-in PROMs.  *OR*  IPOS 7 question F6 equals to ‘Selten’ OR ‘Gar nicht’  *OR*  IPOS 7 question F6 increase of two scale units compared to the previous filled-in PROMs}.  THEN 🡪 ***Information L*** |
| **Look Good Feel Better - Free beauty workshops for cancer patients**  ***Information M***  Look Good Feel Better is a unique, non-profit initiative by leading cosmetics companies in Switzerland, created in close cooperation with hospitals, doctors, and nurses. Free beauty workshops are designed to restore and strengthen the self-confidence and self-esteem of cancer patients. The workshops are conducted by professional beauticians who all work for the foundation on a voluntary basis. The Look Good Feel Better offer is non-medical and completely product- and brand-neutral. | **Preference question**  P7. Are you interested in a free beauty workshop to boost self-esteem and confidence for cancer patients?   - Yes - No | ***Rule M***  IF Preference question P7 equals ‘Ja’.  THEN → ***Information M*** |
| **Nurse counseling**  *Information N*  In addition to the medical consultation hours, we offer specialized care for our patients during office hours. Here, there is space and time for exchange and individual advice in connection with nursing, risks, and psychosocial issues. Nursing professionals support you in self-management and coping with everyday life as part of your illness and treatment. Additionally, a conversation during the nursing consultation should help you get an overview of your appointments and the wide range of counseling and treatment options available at the CCCZ. | All questions from the EORTC QLQ-C30 and the Distress Thermometer. | ***Rule N***  {IF 4 ≤ Distress Thermometer ≤ 6  AND  At least 4 items of EORTC QLQ-C30 equals to or greater than ‘A little bit’.}  OR  Recommended services > 3  THEN 🡪 ***Information N***  **Reference and rational**  As the nurse represents the first line of clinical contact in oncology care, they assume a central role in acting upon data ^11^. |
| **Complementary medicine**  ***Information M.1***  You may find relaxation exercises useful to decrease your stress level [*link to benefits of relaxation exercises*].  ***Information M.2***  At our institute, we offer patients advice on complementary and integrative medicine, along with corresponding treatments. We view people as a whole and coordinate our self-care recommendations and integrative therapy with cancer treatment at the CCCZ. In doing so, we combine current scientific knowledge with many years of experience in complementary medical treatment and take into account the individual situation of the patient. | **Distress Thermometer**  Please circle the number (0-10) that best describes how much stress you have experienced in the past week, including today.  **QLQ C-30 (Rating from 1 = ‘Not at all’ to 4 = ‘Very much’)**  *Emotional Functioning*  *During the past week:*  21. Did you feel tense?  22. Did you worry?  23. Did you feel irritable?  24. Did you feel depressed?  *Pain*  *During the past week:*  9. Have you had pain?  19. Did pain interfere with your daily activities?  *Fatigue*  During the past week:  10. Did you need to rest?  12. Have you felt weak?  18. Were you tired?  *Nausea*  14. Have you felt nauseated? | ***Rule M.1***  IF  {[2 ≤ Distress Thermometer ≤ 4  OR  QLQ C-30 Emotional Functioning (21, 22, 23, 14) equals ‘A little’]  }  THEN → ***Information M.1***  ***Rule M.2***  IF  {QLQ C-30 Pain questions (9, 19), Fatigue questions (10, 12, 18), Nausea (14) equals ‘A little’}  THEN → ***Information M.2***  **Reference and rational**  Evidence symptom management (practice guidelines):   - Pain ^12^ - Fatigue ^13^ - Nausea ^13^ - Anxiety and depression ^14^ |

**Reference**

1. Grassi L, Caruso R, Riba MB, et al. Anxiety and depression in adult cancer patients: ESMO Clinical Practice Guideline. *ESMO Open*. 2023;8(2):101155. doi:10.1016/j.esmoop.2023.101155

2. Donovan KA, Grassi L, McGinty HL, Jacobsen PB. Validation of the Distress Thermometer worldwide: state of the science. *Psychooncology*. 2014;23(3):241-250. doi:10.1002/pon.3430

3. Scotté F, Bossi P, Carola E, et al. Addressing the quality of life needs of older patients with cancer: a SIOG consensus paper and practical guide. *Annals of Oncology*. 2018;29(8):1718-1726. doi:10.1093/annonc/mdy228

4. ElMokhallalati Y, Bradley SH, Chapman E, et al. Identification of patients with potential palliative care needs: A systematic review of screening tools in primary care. *Palliat Med*. 2020;34(8):989-1005. doi:10.1177/0269216320929552

5. Sessa C, Balmaña J, Bober SL, et al. Risk reduction and screening of cancer in hereditary breast-ovarian cancer syndromes: ESMO Clinical Practice Guideline. *Annals of Oncology*. 2023;34(1):33-47. doi:10.1016/j.annonc.2022.10.004

6. Stjepanovic N, Moreira L, Carneiro F, et al. Hereditary gastrointestinal cancers: ESMO Clinical Practice Guidelines for diagnosis, treatment and follow-up†. *Annals of Oncology*. 2019;30(10):1558-1571. doi:10.1093/annonc/mdz233

7. Fabi A, Bhargava R, Fatigoni S, et al. Cancer-related fatigue: ESMO Clinical Practice Guidelines for diagnosis and treatment. *Annals of Oncology*. 2020;31(6):713-723. doi:10.1016/j.annonc.2020.02.016

8. Arends J, Strasser F, Gonella S, et al. Cancer cachexia in adult patients: ESMO Clinical Practice Guidelines☆. *ESMO Open*. 2021;6(3):100092. doi:10.1016/j.esmoop.2021.100092

9. Rauh S, Antonuzzo A, Bossi P, et al. Nutrition in patients with cancer: a new area for medical oncologists? A practising oncologist’s interdisciplinary position paper. *ESMO Open*. 2018;3(4):e000345. doi:10.1136/esmoopen-2018-000345

10. Krech R, Peters S, Kroemer H, et al. Tobacco cessation and the role of ESMO and medical oncologists: addressing the specific needs of cancer patients in times of the COVID-19 pandemic. *ESMO Open*. 2023;8(3):101579. doi:10.1016/j.esmoop.2023.101579

11. Di Maio M, Basch E, Denis F, et al. The role of patient-reported outcome measures in the continuum of cancer clinical care: ESMO Clinical Practice Guideline. *Annals of Oncology*. 2022;33(9):878-892. doi:10.1016/j.annonc.2022.04.007

12. Mao JJ, Ismaila N, Bao T, et al. Integrative Medicine for Pain Management in Oncology: Society for Integrative Oncology–ASCO Guideline. *Journal of Clinical Oncology*. 2022;40(34):3998-4024. doi:10.1200/JCO.22.01357

13. Leitlinienprogramm Onkologie. *S3-Leitlinie Komplementärmedizin in Der Behandlung von Onkologischen PatientInnen* .; 2021.

14. Carlson LE, Ismaila N, Addington EL, et al. Integrative Oncology Care of Symptoms of Anxiety and Depression in Adults With Cancer: Society for Integrative Oncology–ASCO Guideline. *Journal of Clinical Oncology*. 2023;41(28):4562-4591. doi:10.1200/JCO.23.00857
