# Supplementary material for: Collaborative Design and Development of a Patient-Centered Digital Health App for Supportive Cancer Care: Participatory Study
Source: JMIR Hum Factors. 2025 Nov 11;12:e73829. doi: 10.2196/73829 (PMC12648126; doi:10.2196/73829)
Supplement: Multimedia Appendix 4 [file humanfactors_v12i1e73829_app4.docx]

# Multimedia Appendix 4: IT Infrastructure

## 1. System Architecture

The OncoSupport+ app is developed as a web application, meaning users do not need to download a program onto their mobile devices, but rather visit a website using their preferred browser. This solution has the benefit that the additional step of downloading and installing additional software simplifies the onboarding process for our users. Similarly, designing a web application allows us to use modern web technologies to implement a design that can be accessed through a large variety of devices (and more importantly screen sizes) using responsive design patterns.

OncoSupport+ uses three main components to achieve its goals: (1) a React-based frontend which the users directly interact with, (2) a FastAPI-based backend facilitating the communication between the frontend and our storage layer and (3) a PostgreSQL database where all our data is stored. For easier deployment and network isolation, we have decided to deploy these three components through Docker. If so desired, the architecture could be extended to include two separate database instances such that the user-identifying data is physically kept separate from the questions.


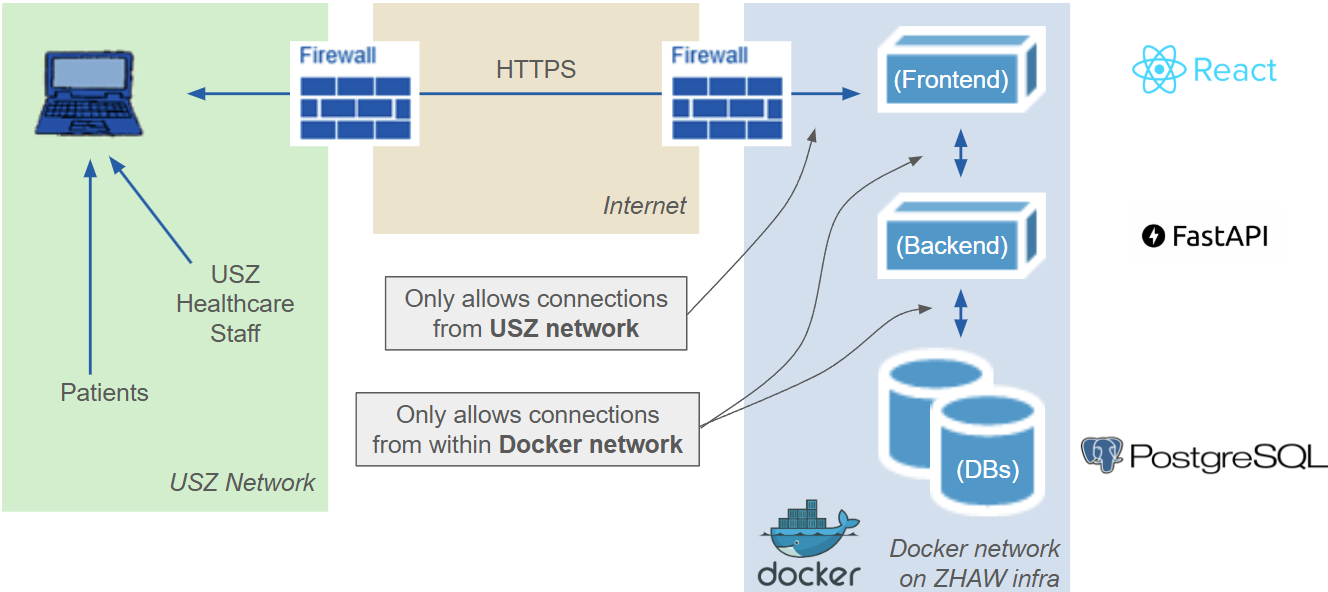


Figure D1: Visualization of the communication workflow in OncoSupport+

## 2. Hosting

Developing a web application for hospital use brings additional risk factors, especially in terms of security. Where and how the application is hosted thus becomes an important choice in reducing potential risks. During the development, we have been in contact with the IT department of USZ to ensure that it can be integrated into the ecosphere of the hospital at a later date but decided to develop and host the web application – at least initially – on the infrastructure provided by ZHAW. While we are aware that hosting it as part of the hospital infrastructure from the start would be beneficial for security reasons, the reality is that one has to prove that a novel third party application is beneficial first before significant hospital resources are leveraged. This further emphasizes the importance to get and stay in contact with the relevant people throughout the lifetime of the project, as not to create an application that is technically doomed to be adopted later.

## 3. Data Security & Privacy

The University Hospital Zurich (USZ) as the name implies is located in Zurich, Switzerland. As such, the primary laws and regulations are the Swiss Federal Act on Data Protection and the General Data Protection Regulation (GDPR) of the European Union (EU), since it is reasonably likely that patients could be citizens of the EU.

To protect our users’ data (and especially the sensitive patient data), we have taken the following measures:

**User Consent and Transparency**:

- Before patients start using OncoSupport+, they are required to read and sign an Informed Consent form which outlines the aim of the study, what data is collected and how it is used.

**Data Access and User Control**

- Patients can view their own data and update or delete information, where applicable.
- Access control is implemented in such a way that patients can only see their own information, while healthcare professionals can see the data of all patients.
- When registering as a new user in the OncoSupport+ app, users are registered as patients. The higher access of healthcare professionals is only granted to actual professionals working at the hospital after manual checks and only by administrators.
